# Farnesane-Type Sesquiterpenoids with Antibiotic Activity from *Chiliadenus lopadusanus*

**DOI:** 10.3390/antibiotics10020148

**Published:** 2021-02-02

**Authors:** Marco Masi, Emanuela Roscetto, Alessio Cimmino, Maria Rosaria Catania, Giuseppe Surico, Antonio Evidente

**Affiliations:** 1 Dipartimento di Scienze Chimiche, Università di Napoli Federico II, Complesso Universitario Monte S. Angelo, via Cintia 4, 80126 Napoli, Italy; marco.masi@unina.it (M.M.); evidente@unina.it (A.E.); 2 Dipartimento di Medicina Molecolare e Biotecnologie Mediche, Università di Napoli Federico II, via Pansini 5, 80131 Napoli, Italy; emanuela.roscetto@unina.it (E.R.); mariarosaria.catania@unina.it (M.R.C.); 3 Dipartimento di Scienze e Tecnologie Agrarie, Alimentari, Ambientali e Forestali, Sez. Patologia Vegetale ed Entomologia, Università di Firenze, Piazzale delle Cascine 28, 50144 Firenze, Italy; giuseppe.surico@unifi.it

**Keywords:** *Chiliadenus lopadusanus*, sesquiterpenes, antibacterial activity, antibiofilm activity

## Abstract

*Chiliadenus lopadusanus* Brullo is an Asteraceae plant species endemic to Lampedusa island, the largest island of the Pelage archipelago, Italy. The organic extract of its whole aerial parts, showing antibiotic activity against *Staphylococcus aureus* and *Acinetobacter baumannii*, wasfractionated employing bioguided purification procedures affording three main farnesane-type sesquiterpenoids. They were identified by spectroscopic methods (NMR and ESIMS data) as the (*E*)-3,7,11-trimethyldodeca-1,6,10-triene-3,9-diol, (*E*)-10-hydroxy-2,6,10-trimethyldodeca-2,6,11- trien-4-one and (*E*)-10-hydroxy-2,6,10-trimethyl-dodeca-6,11-dien-4-one, commonly named 9-hydroxynerolidol, 9-oxonerolidol, and chiliadenol B, respectively. These three sesquiterpenes, isolated for the first time from *C. lopadusanus*, were tested on methicillin-resistant *S. aureus* and *A. baumannii* showing antibacterial and antibiofilm activities. This plant could be used as a source to isolate secondary metabolites as potential new antibiotics.

## 1. Introduction

Antimicrobial resistance (AMR) is a phenomenon that seriously endangers the control of diseases around the world [1]. Microorganisms resistant to antimicrobials, including multidrug-resistant ones, are often the cause of healthcare-associated infections but also infections of outpatients. In addition, they can be present in the normal bacterial flora of all healthy people, in companion animals and in the environment [2,3,4]. Resistant microorganisms are also present in food-producing animals, and sometimes even in food [5].

Until now, the threat of bacterial resistance to antibiotics has been underestimated. However, antibiotic resistance is now a phenomenon recognized all over the world as a priority in the health sector due to the important implications both from a clinical point of view (longer hospital stays, higher mortality) and from an economic point of view (rising costs) [6,7]. 

The World Health Organization (WHO) is sounding the alarm on the AMR phenomenon which in recent decades has practically rendered most of the available antibiotics useless, including the most recent carbapenem drugs associated with innovative inhibitors of resistance enzymes. In fact, both Gram-positive and Gram-negative pathogens such as, for example, the ESKAPE group (*Enterococcus faecium*, *Staphylococcus aureus*, *Klebsiella pneumoniae*, *Acinetobacter baumannii*, *Pseudomonas aeruginosa*, *Enterobacter* spp.) are multiresistant. Therefore, the clinical efficacy of many classes of antibiotics is compromised with a consequent increase in mortality associated with infections [8,9]. The WHO has also estimated that about 80% of chronic infections are related to the formation of biofilms. Biofilms are defined as communities of properly organized microorganisms attached to an inert or living substrate and embedded in a self-produced extracellular exopolysaccharidic matrix. Biofilm formation is regarded as the most important virulence factor protecting the sessile bacteria against antibacterial compounds and host immune responses [10]. Biofilm-related infections represent a major global problem in the hospital setting due to their intrinsic recalcitrance toward antibiotics and to difficulties in treatment.

Unfortunately, the development of new antibiotics proceeds very slowly, making it necessary to search for new potential antimicrobial scaffolds from different sources [11,12,13,14,15]. Herbal medicine, according to the WHO classification, belongs to the vast system of traditional medicine. Many populations living in poor countries and especially in rural areas, consider the world of plants as the main pivot for their primary health, relying on the beneficial properties of trees, herbs, and fruits to cure certain diseases and heal wounds [16,17,18].

Plants are a rich reservoir of compounds with several biological activities, including antimicrobial properties [19,20,21,22,23]. Plant extracts can have a good activity on their own or can be sources of antimicrobial compounds effective against pathogens responsible for infections, which are difficult to treat also for their ability to form biofilms [24,25,26].

**Figure 1:**
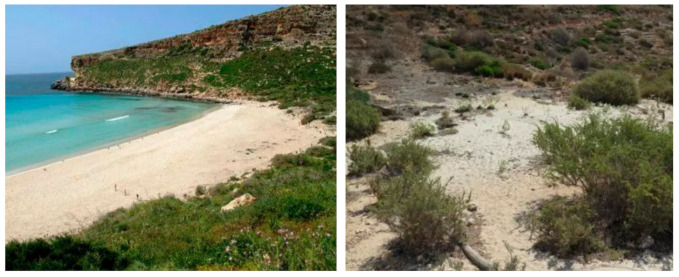
*Chiliadenus lopadusanus* in Conigli bay, Lampedusa island, Italy.

A screening on endemic plants collected in different regions of the Mediterranean basin was aimed to find new antibiotics. Among the corresponding organic extracts, that of *Chiliadenus lopadusanus* showed a growth inhibiting activity against some human pathogenic bacteria. *C. lopadusanus* Brullo (Figure 1) is an Asteraceae endemic species growing spontaneously on Lampedusa island, the largest island of the Pelage archipelago, about 100 km from the North Africa coast and 200 km from the Sicily coast, Italy [27]. 

**Figure 2:**
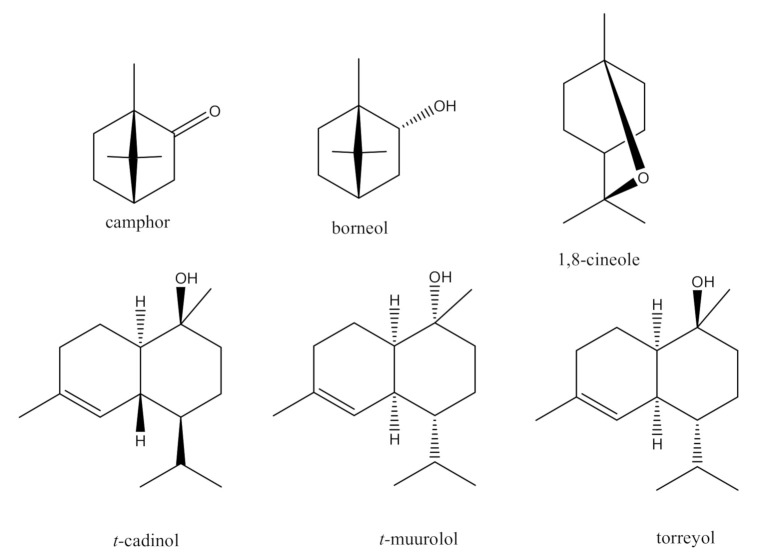
The chemical structures of camphor, borneol, 1,8-cineole, *t*-cadinol, *t*-muurolol, and torreyol.

The essential oils produced by the leaves and flowers of *C. lopadusanus* were first investigated by Sacco and Maffei [28] using GC and GC-MS spectrometry and camphor, borneol, and 1,8-cineole (Figure 2) were identified as the major constituents. More recently, the essential oils of the same plant were extracted by hydrodistillation and analyzed by GC-MS leading to the identification of 98 compounds [27]. Camphor was identified as the main constituent together with the monoterpene 1,8-cineole and the sesquiterpenes *t*-cadinol, *t*-muurolol, and torreyol (Figure 2). These terpenes are reported in the literature to play a role in the potential spasmolytic, antibacterial, antifungal, and allelopathic activity [28,29,30]. However, complete chemical studies of all the major metabolites of *C. lopadusanus* to characterize their biological activity have not been performed.

In fact, the chemical investigation of the secondary metabolites produced by the plants belonging to the genus *Chiliadenus* (*Asteraceae* family) is very limited. The components of the essential oil obtained from leaves extract of Spanish wild growing *Chiliadenus glutinosa* were analyzed for the first time by GC-MS and GC [31]. Several terpenoid derivatives were identified including camphor and borneol as the main components. Successively, a comparison of the essential oil extracted with direct thermal desorption techniques was reported [32]. The essential oil obtained from *Chiliadenus iphionoides*, a plant endemic to the eastern Mediterranean area and growing wild in Jordan was analyzed by Avato et al. [33]. Borneol and its acetyl and formyl esters with a potential antispasmodic activity were identified as the major constituents by GC and GC—MS [33]. Eight new compounds and eight previously identified metabolites were instead isolated from *Chiliadenus montanus*, an hearb endogeneous of the North Sinai region of Egypt. Their antimicrobial activity was evaluated against a panel of bacterial and fungal strains [34].

Therefore, this manuscript reports the extraction, bioguided purification, and the chemical characterization by spectroscopic methods of the main metabolites produced by *C. lopadusanus*. Antibacterial and antibiofilm activities of the isolated metabolites were assayed against *Staphylococcus aureus* and *Acinetobacter baumannii*, nosocomial pathogenic bacteria responsible for severe infections.

## 2. Results and Discussion

### 2.1. Isolation and Identification of Bio-Active Metabolites

**Figure 3:**
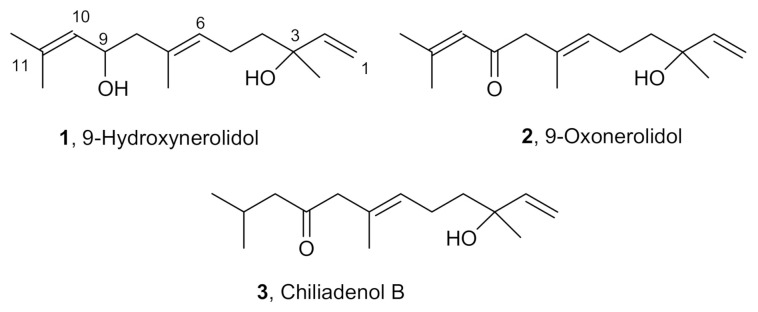
Structures of 9-hydroxynerolidol, 9-oxonerolidol, and chiliadenol B (**1**–**3**).

The *n*-hexane extract of the whole aerial parts of *C. lopadusanus* was chromatographed, as reported in detail in the Materials and Methods section, to afford (*E*)-3,7,11-trimethyldodeca-1,6,10-triene-3,9-diol, (*E*)-10-hydroxy-2,6,10-trimethyldodeca- 2,6,11-trien-4-one, and (*E*)-10-hydroxy-2,6,10-trimethyl-dodeca-6,11-dien-4-one, commonly named 9-hydroxynerolidol, 9-oxonerolidol, and chiliadenol B (compounds **1**–**3**, Figure 3). 

Compounds **1**–**3** were identified comparing their physics (specific rotation) spectroscopic (mono and bidimensional NMR spectra and ESIMS) with data partially reported in the literature and in particular, for 9-hydroxynerolidol and 9-oxonerolidol (**1** and **2**) with data reported by Stoessl et al. [33] and for chiliadenol B (**3**) with those reported by Hegazy et al. [34]. Therefore, in Tables S1–S3, the chemical shifts of all the protons and carbons of the three sesquiterpenoids (**1**–**3**) were unambiguously reported with some updated values with respect to the ones already reported [34,35].

This is the first isolation of farnesane-type sesquiterpenoids **1**–**3** (Figure 3) from *C. lopadusanus*. 9-Hydroxynerolidol (**1**) was first isolated as a stress metabolite from *Solanum melongena* [35] together with 9-oxonerolidol (**2**) which was preliminarily isolated also from the leaf oil of the camphor tree by Hiroi and Takaoka [36]. Chiliadenol B (**3**) was isolated for the first time from the Egyptian herbal medicine *Chiliadenus montanus* [34]. 

### 2.2. Antibacterial and Anti-Biofilm Activities

The organic extract of *C. lopadusanus* was tested for its antibacterial activity against the methicillin-resistant reference strain *Staphylococcus aureus* ATCC43300 and reference strain *Acinetobacter baumannii* ATCC747. At the concentration of 500 μg/mL, the *n*-hexane extract showed a total inhibition of planktonic growth of both strains (Table 1).

The secondary metabolites **1**, **2**, **3** obtained from the extract, were tested individually against the reported strains identifying the minimal inhibitory concentration (MIC) and minimal bactericidal concentration (MBC) values. Both compounds **1** and **2** showed antibacterial activity: Compound **1** exhibited MIC values of 150 μg/mL on the *A. baumannii* strain and 75 μg/mL on the *S. aureus* strain, while compound **2** showed a MIC value of 150 μg/mL for both test strains (Table 1). The MBC values of compounds **1** and **2** were higher than 300 μg/mL for both tested strains. No antimicrobial activity on the planktonic bacterial growth was detected for substance **3**. 

These results are in agreement with those of Hegazy et al. [34] who previously tested chiliadenol B on a different panel of bacterial species and did not show any antimicrobial activity. The ability to inhibit the bacterial growth has been documented for several terpenes and terpenoid derivatives [37,38,39]. The antibacterial activity is most frequently explained by their ability to destroy the plasma membrane integrity [40], but it has also been hypothesized that they can inhibit efflux pumps or ATPases on the bacterial membranes [41]. In this large group of phytochemicals, it is known that sesquiterpenoid compounds also have antimicrobial activity. Gomes et al. [42] showed that farnesol was able to inhibit *Staphylococcus epidermidis* planktonic cells. Structurally diverse halosesquiterpenoids from *Laurencia composita* Yamada showed an antibacterial effect against *S. aureus* [43]. However, the antibacterial activity of sesquiterpenoids **1** and **2** from *C. lopadosanus* has not been previously described. Interestingly, the structural differences between these two compounds and chiliadenol B are related to a clear difference in antimicrobial activity. 

In fact, the two compounds **1** and **2** are structurally related and differed only in the presence of the hydroxyl group at C-9 in **1**, which is oxidized in **2**. In **3**, the double bond between C-10 and C-11 was absent. The α,β-unsaturated carbonyl group present in **1** could be responsible for its antibiotic activity as probably it is a site for nucleophile Michael addition, as frequently reported for other natural bio-active metabolites [44,45]. Similarly, the mode of action could be invoked for **2** as the hydroxyl group at C-9 could be easily oxidized within the cells as given by the more stable α,β-unsaturated carbonyl group as in **1**. This does not occur in **3** as it lacks the double bond to be conjugated with the carbonyl at C-9.

Noteworthy, Li et al. [46] reported that (3*S*)-(+)-9-oxonerolidol from *Cinnamomum camphora* (L.) J. Presl showed an in vitro anti-inflammatory activity by inhibiting the nuclear factor κB (NF-κB). However, its absolute configuration was only assigned by comparison of its specific optical rotation with that previously reported for the same sesquiterpene [35]. This assignment requires confirmation using chiroptical and computational methods. The simultaneous presence of antibacterial and anti-inflammatory activity in the same compound can be very useful as the inflammatory response that represents the early defense against the antibacterial infection can at the same time contribute to tissue damage.

**Figure 4:**
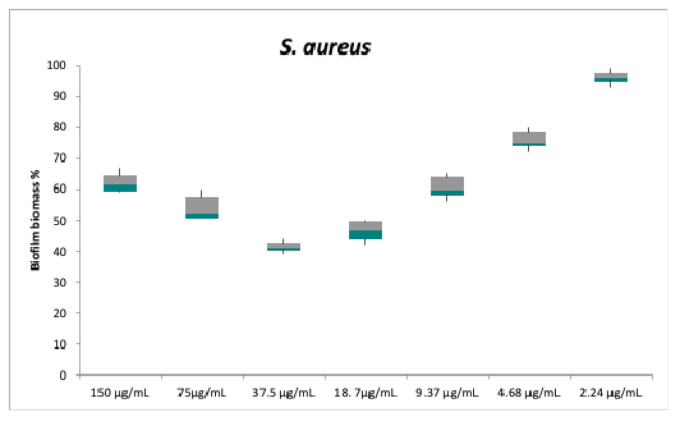
In vitro biofilm formation of *S. aureus* following an overnight treatment with serial dilutions of compound **3**. Biofilm formation was determined by the cristal violet assay. Biofilm biomass values are presented as the mean percentage ± standard deviation (SD) and *S. aureus* biofilm biomass without a treatment is assumed to be 100%. Each pair of means was compared using a Tukey’s multiple comparisons test; no significant differences were detected between the following pairs: 150℃9.37 μg/mL; 37.5℃18.7 μg/mL.

**Figure 5:**
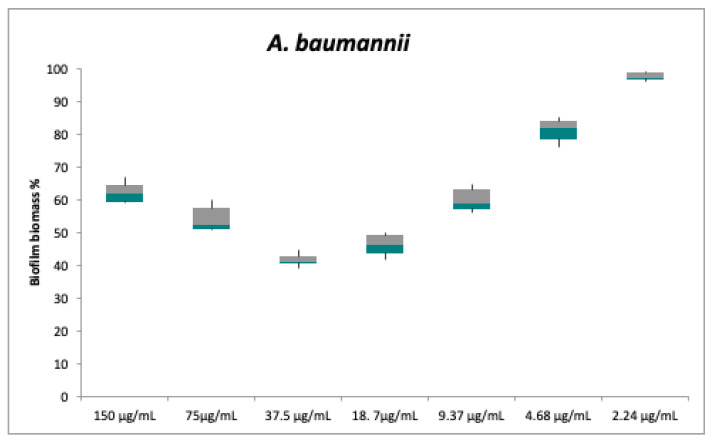
In vitro biofilm formation of *A. baumannii* following an overnight treatment with serial dilutions of compound **3**. Biofilm formation was determined by the cristal violet assay. Biofilm biomass values are presented as the mean percentage ± SD and *A. baumannii* biofilm biomass without a treatment is assumed to be 100%. Each pair of means was compared using a Tukey’s multiple comparisons test; no significant differences were detected between the following pairs: 150℃9.37 μg/mL; 37.5℃18.7 μg/mL.

Regarding the anti-biofilm activity, compounds **1** and **2** do not appear to be effective. Despite the lack of antibacterial activity against planktonic cells, compound **3** was instead able to inhibit the biofilm formation of both strains: The test strains were cultured in multiwells in the biofilm growth mode in the presence of different concentrations of compound **3** (between 2.24 to 150 μg/mL). Biofilm formation by *S. aureus* and *A. baumannii* was already weakly inhibited at the concentration of 9.37 μg/mL (Figure 4 and Figure 5). 

The concentration value of 18 μg/mL caused a biofilm inhibition of 53 ± 3.5% for *S. aureus* and 54 ± 3.3% for *A. baumannii*. For both bacterial strains, the maximum inhibition was obtained at 37.5 μg/mL, a concentration that caused nearly 60% reduction in the biofilm mass (59 ± 1.7% and 58.5 ± 2% inhibition for *S. aureus* and *A. baumannii*, respectively), despite the lack of statistically significant difference compared to the previous dose.

At concentrations greater than 37.5 μg/mL, the biofilm formation gradually resumes increasing for both test strains. High concentrations probably allow the formation of intra- or inter-molecular hydrogen bond between the hydroxyl group at C-3 and the carbonyl at C-9 in **3**. However, this hypothesis needs to be confirmed. 

Compound **3**, as above reported, differed from both **1** and **2**, for lack of the double bound between C-10 and C-11. Consequently, a nucleophile Michael addiction to the *α*, *β*-unsaturated carbonyl group as in **1** and **2** could be not possible. A different mechanism of action of **3** can be suggested with respect to that of **1** and **2**. This structural factor confers to compound **3** a greater conformational freedom than **1** and **2,** as also observed by an inspection of a Drieding model. 

Biofilm formation is a complex process that takes place through several stages: Adhesion of bacterial cell to a surface, cell multiplication, production of the extracellular matrix, and dispersal. Communication between microbial cells through the quorum sensing system is critical during biofilm formation. Quorum sensing also regulates the expression of virulence genes and the metabolic activity of bacterial cells [47]. Rukayadi et al. [48] showed that xanthorrhizol, a bisabolane-type sesquiterpenoid extracted from *Curcuma xanthorrhiza*, inhibited biofilm formation by *Streptococcus mutans* in the adhesion phase. Alves et al. [49] reported that linalool, a monoterpenoid present in the essential oil of *Coriandrum sativum*, showed anti-biofilm activity against *A. baumanni* by inhibiting bacterial adhesion and interfering with the quorum-sensing system. T-farnesol, a natural sesquiterpenoid alcohol found in propolis, was able to markedly reduce the bacterial viability and production of extracellular insoluble polysaccharide in the *S. mutans* biofilm [50]. Farnesol was also effective in reducing the average thickness and substrate coverage of *Staphylococcus epidermidis* biofilm, even in the case of quorum sensing mutants [51]. Further studies will be useful to understand if compound **3** interferes with the adhesion, growth, cell-to-cell communication, or maturation phase of the biofilm.

The structural differences between the three compounds could determine a different interaction with components of the bacterial surface or the intercellular matrix and explain their different antibiofilm activities. Therefore, structure-function correlation studies may suggest modifications to these skeletons to enhance antibacterial activity. 

## 3. Materials and Methods 

### 3.1. General Experimental Procedures

^1^H and ^13^C nuclear magnetic resonance (NMR) spectra were recorded at 400 and 100 MHz, respectively, in CDCl_3_ by Bruker spectrometers (Karlsruhe, Germany). The same solvent was used as an internal standard. Carbon multiplicities were determined using distortions enhancement by polarization transfer (DEPT) spectra [52]. DEPT, correlation spectroscopy with a 45° mixing pulse (COSY-45), heteronuclear single quantum correlation (HSQC), and heteronuclear multiple bond correlation (HMBC) experiments [52] were performed using Bruker microprograms. Electrospray ionization mass spectra (ESIMS) and liquid chromatography/mass spectrometry (LC/MS) analyses were carried out using the LC/MS TOF system (Agilent 6230B, HPLC 1260 Infinity) (Milan, Italy). The high-performance liquid chromatography (HPLC) separation was performed using a Phenomenex LUNA (C18 (2) 5 u 150 × 4.6 mm) (Torrance, CA, USA). Analytical, preparative, and reverse phase thin-layer chromatography (TLC) were carried out on silica gel (Merck, Kieselgel 60, F_254_, 0.25, 0.5 mm, and RP-18 F_254_s, respectively) plates (Merck, Darmstadt, Germany). The spots were visualized by exposure to ultraviolet (UV) radiation, or by spraying first with 10% H_2_SO_4_ in MeOH, and then with 5% phosphomolybdic acid in EtOH, followed by heating at 110 °C for 10 min. Column chromatography (CC) was performed using silica gel (Kieselgel 60, 0.063–0.200 mm) (Merck, Darmstadt, Germany). All the solvents were supplied by Sigma-Aldrich (Milan, Italy).

### 3.2. Plant Material

Whole aerial parts of *Chiliadenus lopadusanus* plants were collected fresh at a pre-flowering stage in May 2019 in Lampedusa island (Italy) by Mr. Fabio Giovanetti and identified by Prof. G. Surico, University of Florence, Italy. The plant specimen is deposited in the collection of Department of Agriculture, Food, Environment, and Forestry (DAGRI), Section of Agricultural Microbiology, Plant Pathology and Enthomology, University of Florence, Italy, n. DAGRI-56. The air-dried plant material was then grinded to obtain a tiny powder using a laboratory mill and packaged in plastic bags under a vacuum until its use.

### 3.3. Isolation of Fungal Metabolites

**Figure 6:**
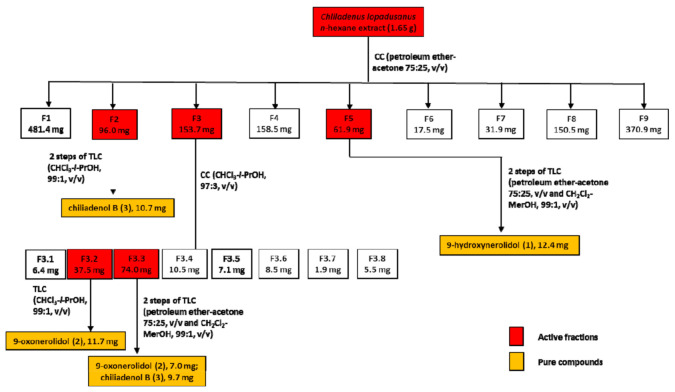
Chromatographic purification diagram of the metabolites extracted from *C. lopadusanus*.

Plant material (450 g) was extracted (1 × 1 L) by H_2_O/MeOH (1/1, *v*/*v*) under stirred conditions at room temperature for 24 h, the suspension centrifuged and the supernatant extracted by *n*-hexane (3 × 400 mL) and successively with CH_2_Cl_2_ (3 × 400 mL). The residue (1.65 g) of *n*-hexane organic extract, showing interesting antibacterial activity against *S. aureus* and *A. baumannii*, was purified by CC eluted with petroleum ether/acetone (75/25, *v*/*v*), yielding nine groups of homogeneous fractions. Among them, the residues of fractions F2, F3, and F5 retained an antibiotic activity and were further purified using different steps of CC and TLC, as shown in Figure 6. 

F2 was further purified by two TLC steps giving a homogeneous compound identified as chiliadenol B (**3**, Rf 0.65, 10.7 mg). F3 was further purified by CC yielding eight groups of homogeneous fractions. Among them, only fractions F3.2 and F3.3 retain the activity. The purification of F3.2 by TLC gave a homogeneous compound identified as 9-oxonerolidol (**2**, Rf 0.65, 11.7 mg). F.3.3 was further purified through two TLC steps giving a further amount of chiliadenol B (**3**, 9.7 mg) and of 9-oxonerolidol (**2**, 7.0 mg). The residue of F5 of the original column was further purified by two TLC steps giving a pure oil identified as 9-hydroxynerolidol (**1**, Rf 0.65, 12.4 mg).

#### 3.3.1. 9-Hydroxynerolidol (**1**)

9-Hydroxynerolidol (**1**): [α]^25^_D_ + 5.2 (c 0.04, CHCl_3_) (lit. [33]: [α]^25^_D_ + 0 (5 mg/mL, EtOH); ^1^H NMR (see Table S1), δ (*J* in Hz): 5.93 (dd, 17.2 and 10.7, H-2), 5.29 (br t, 7.3, H-6), 5.23 (dd, 17.2 and 1.2, H-1A), 5.17 (br dd, 8.5 and 1.3, H-10), 5.08 (dd, 10.7 and 1.2, H-1B), 4.66 (ddd, 11.8, 8.5 and 2.7, H-9), 2.10 (2H, m, H-4), 2.02 (2H, m, H-8), 1.74 (3H, s, Me-12), 1.71 (3H, s, Me-13), 1.68 (3H, s, Me-14), 1.60 (2H, m, H-5), 1.30 (s, Me-15); ^13^C NMR (see Table S1), δ: 144.9 (d, C-2), 134.6 (s, C-7), 131.8 (s, C-11), 128.4 (d, C-10), 127.5 (d, C-6), 111.7 (t, C-1), 73.4 (s, C-3), 66.0 (d, C-9), 48.1 (t, C-8), 41.8 (t, C-4), 27.9 (q, C-15), 25.7 (q, Me-12), 22.9 (t, C-5), 18.1 (q, Me-13), 16.2 (q, Me-14); ESIMS (+), *m*/*z* 221 [M − H_2_O + H]^+^, 239 [M + H]^+^.

#### 3.3.2. 9-Oxoxynerolidol (**2**)

9-Oxoxynerolidol (**2**): [α]^25^_D_ + 14.4 (c 0.04, CHCl_3_) (lit. [34]: [α]^25^_D_ + 15.38 (CCl_4_); (lit. Stoessl et al., 1975) [α]^25^_D_ + 24 (c 1.0, CCl_4_); ^1^H NMR (see Table S2), δ (*J* in Hz): 6.11 (s, H-10), 5.94 (dd, 17.5 and 10.5, H-2), 5.29 (br t, 7.0, H-6), 5.26 (br d, 17.5, H-1A), 5.09 (br d, 10.5, H-1B), 3.06 (2H, s, H-8), 2.16 (3H, s, Me-13), 2.10 (2H, m, H-5), 1.90 (3H, s, Me-12), 1.63 (3H, s, Me-14), 1.62 (3H, m, H-4), 1.31 (3H, s, Me-15); ^13^C NMR (see Table S2), δ: 199.2 (s, C-9), 155.7 (s, C-11), 144.5 (d, C-2), 129.9 (s, C-7), 129.1 (d, C-10), 122.4 (d, C-6), 111.5 (t, C-1), 73.0 (s, C-3), 55.3 (t, C-8), 41.3 (t, C-4), 27.9 (q, Me-15), 27.5 (q, Me-12), 22.9 (t, C-5), 20.4 (q, Me-13), 16.3 (q, Me-14); ESIMS (+), *m*/*z* 219 [M − H_2_O + H]^+^, 237 [M + H]^+^.

#### 3.3.3. Chiliadenol B (**3**)

Chiliadenol B (**3**): [α]^25^_D_ + 4.0 (c 0.04, CHCl_3_) (lit. [28]: [α]^25^_D_ + 3.67 (c 0.03, CHCl_3_); ^1^H NMR (see Table S3), δ (*J* in Hz): 5.94 (dd, 17.3 and 10.7, H-2), 5.28 (t, 7.3, H-6), 5.26 (br d, 17.3, H-1A), 5.10 (br d, 10.7, H-1B), 3.04 (2H, s, H-8), 2.30 (2H, d, 6.7, H-10), 2.14 (m, H-11), 2.10 (2H, m, H-5), 1.67 (2H, m, H-4), 1.62 (3H, s, Me-14), 1.31 (3H, s, Me-15), 0.93 (3H, br s, Me-12), 0.91 (3H, br s, Me-13); ^13^C NMR (see Table S3), δ: 209.4 (s, C-9), 144.8 (d, C-2), 129.4 (d, C-6), 128.9 (s, C-7), 73.0 (s, C-3), 54.0 (t, C-8), 50.4 (t, C-10), 41.4 (t, C-4), 27.9 (q, Me-15), 24.2 (t, C-11), 22.7 (t, C-5), 22.6 (q, overlapped signals of Me-12 and Me-13), 16.3 (q, Me-14); ESIMS (+), *m*/*z* 221 [M − H_2_O + H]^+^, 239 [M + H]^+^.

### 3.4. Test Bacterial Strains and Culture Conditions

Bacterial strains used in this study were methicillin-resistant *Staphylococcus aureus* ATCC 43,300, and *Acinetobacter baumannii* ATCC 747. All strains were stored as 15% (*v/v*) glycerol stocks at −80 °C. Before each experiment, cells were sub-cultured from the stocks onto tryptic soy agar (TSA; Becton Dickinson, Franklin Lakes, NJ, USA) plates at 37 °C for 24 h. The identification and antibiotic susceptibility profile of the reference bacteria were performed using the Vitek II (bioMérieux, Marcy-l’Étoile, France) and Phoenix (Becton Dickinson) systems. 

### 3.5. Antimicrobial Assays

The antibacterial activity of plant extract and secondary metabolites was assayed on test strains by a standard broth micro-dilution method in 96-well polystyrene plates using Mueller-Hinton Broth 2 (MHB2). For each strain, starting from bacterial suspensions with a turbidity of 0.5 McFarland (corresponding to 1–5 × 10^8^ cells/mL) and subsequently adjusted to approximately 5 × 10^6^ CFU/mL, 100 μL aliquots of these bacterial suspensions were dispensed in all the wells. Then, the activity of the extract was tested by adding 100 µL of a 500 µg/mL solution to the wells, while to test the three metabolites the wells were added with 100 μL of two-fold dilutions starting from 300 µg/mL. The wells without substances were used as a positive growth control. Conventional antibiotics, selected depending on antibiotic-susceptibility profiles of the test strains, were included as the control: Colistin (ranged from 0.2 to 12.5 µg/mL) was used for *A. baumannii* and teicoplanin (ranged from 0.5 to 4 µg/mL) for *S. aureus*. Plates were incubated at 37 °C for 19 h under shaking (300 rpm). Then, the medium turbidity was measured by a spectrophotometer at 595 nm (Bio-Rad Laboratories Srl, Hercules, CA, USA). The antimicrobial activity was expressed as a percentage of microbial growth inhibition. 

The minimal inhibitory concentration (MIC) and minimal bactericidal concentration (MBC) of secondary metabolites were determined: The MIC was defined as the lowest concentration of metabolite that caused no visible bacterial growth in the wells. The MBC was defined as the lowest compound concentration that yields no microbial growth on the agar plates of each sample, previously treated with compound concentrations equal to or higher than the MIC. The extract and each metabolite were tested in triplicate and each experiment was performed twice. To be sure that the 2% of dimethyl sulfoxide (DMSO; Sigma-Aldrich, St. Louis, MO, USA) present in the 2× stock solutions of the metabolites did not act on the bacterial growth, the effect of serial dilutions of DMSO starting from 1% on the growth of the test strains was separately tested.

### 3.6. Biofilm Formation Inhibition Assay

The total biomass of the biofilm formed in vitro in the presence of the test compounds was analyzed using the crystal violet (CV) staining method in flat-bottomed 96-well microplates, as described by Stepanović et al. [53]. For each strain, a cell suspension in MHB2 supplemented with 10% (*w*/*v*) glucose was prepared and diluted to obtain a suspension of 1 × 10^6^ CFU/mL. One hundred microliters of this suspension were incubated with 100 µL of serial dilutions of sub-MIC concentrations in MHB2 of the compounds test. The negative control was represented by the microbial suspension inactivated by boiling. The positive controls were compound-free wells. The microtiter was incubated at 37 °C for 19 h. The non-adherent cells were then removed with gentle aspiration and gentle washings with a phosphate-buffered saline (PBS; Sigma-Aldrich) and the biofilm was dried at 60 °C for 1 h and subsequently stained with a 0.1% (*w*/*v*) crystal violet solution for 30 min.

After washing with PBS and solubilization with absolute ethanol to release the dye from the biofilm, the spectrophotometric reading is performed at 570 nm. The absorbance recorded was correlated to the amount of biofilm produced. The percentage of biofilm mass reduction was calculated using the formula: [(Ac − At)/Ac] × 100, where Ac is the OD570 for control wells and At is the OD570 in the presence of the tested compound.

### 3.7. Statistical Analysis

Statistical analyses of biofilm inhibition data were carried out using the ordinary one-way analysis of variance (ANOVA) and Tukey’s multiple comparisons test. The box plots shown in this study were built by the QI Macros software and the results were presented as the mean ± standard deviation. Differences with a *p* < 0.005 were considered statistically significant.

## 4. Conclusions

Antibiotic resistance poses a major threat to human health. In medicine, plants have been used for millennia as a treatment for human diseases and can represent a source of many antibacterial compounds. For the first time, our study reports the isolation of the three sesquiterpenes, namely 9-hydroxynerolidol, 9-oxonerolidol, and chiliadenol B, from the plant *C. lopadusanus* Brullo. The antimicrobial activity of compounds 9-hydroxynerolidol and 9-oxonerolidol, as well as the anti-biofilm activity of chiliadenol B were also reported. Therefore, it would be of considerable interest to evaluate the possible use of a combination of the three compounds for the prevention of biofilm-related infections.

## Figures and Tables

**Table 1:** Antibacterial activity of the leaves extract, expressed as a percentage of growth inhibition, and of compounds **1**–**3**, expressed as MIC (μg/mL) ± standard deviation (SD), against test strains.

Strains	Plant Extract (500 µg/mL)	Compound 1 MIC	Compound 2 MIC	Compound 3 MIC	CO MIC	TE MIC
*S. aureus*	100% inhibition	75 ± 1.8	150 ± 3.8	nd	-	1 ± 0.1
*A. baumannii*	100% inhibition	150 ± 4.2	150 ± 3.2	nd	0.78 ± 0.0	-

Colistin (CO) and teicoplanin (TE) were used as positive controls; nd: not detected.

## Data Availability

Data are contained within the text.

## Supplementary Materials

The following are available online at https://www.mdpi.com/2079-6382/10/2/148/s1. Figure S1. ^1^H NMR spectrum of 9-hydroxynerolidol, **1** (CDCl_3_, 400 MHz); Figure S2. ^13^C NMR spectrum of 9-hydroxynerolidol, **1** (CDCl_3_, 100 MHz). Figure S3. COSY spectrum of 9-hydroxynerolidol, **1** (CDCl_3_, 400 MHz); Figure S4. HSQC spectrum of 9-hydroxynerolidol, **1** (CHCl_3_, 400/100 MHz); Figure S5. HMBC spectrum of 9-hydroxynerolidol, **1** (CHCl_3_, 400/100 MHz); Figure S6. NOESY spectrum of 9-hydroxynerolidol, **1** (CDCl_3_, 400 MHz). Figure S7. ESIMS spectrum of 9-hydroxynerolidol, **1**, recorded in positive modality; Figure S8. ^1^H NMR spectrum of 9-oxonerolidol, **2** (CDCl_3_, 400 MHz); Figure S9. ^13^C NMR spectrum of 9-oxonerolidol, **2** (CDCl_3_, 100 MHz). Figure S10. COSY spectrum of 9-oxonerolidol, **2** (CDCl_3_, 400 MHz); Figure S11. HSQC spectrum of 9-oxonerolidol, **2** (CDCl_3_, 400/100 MHz); Figure S12. HMBC spectrum of 9-oxonerolidol, **2** (CDCl_3_, 400/100 MHz). Figure S13. NOESY spectrum of 9-oxonerolidol, **2** (CDCl_3_, 400 MHz); Figure S14. ESIMS spectrum of 9-oxonerolidol, **2**, recorded in positive modality; Figure S15. ^1^H NMR spectrum of chiliadenol B, **3** (CDCl_3_, 400 MHz); Figure S16. ^13^C NMR spectrum of chiliadenol B, **3** (CDCl_3_, 100 MHz); Figure S17. COSY spectrum of chiliadenol B, **3** (CDCl_3_, 400 MHz); Figure S18. HSQC spectrum of chiliadenol B, **3** (CDCl_3_, 400/100 MHz); Figure S19. HMBC spectrum of chiliadenol B, **3** (CDCl_3_, 400/100 MHz); Figure S20. NOESY spectrum of chiliadenol B, **3** (CDCl_3_, 400 MHz); Figure S21. ESIMS spectrum of chiliadenol B, **3** recorded in positive modality. Table S1. ^1^H and ^13^C NMR data of 9-hydroxynerolidol (**1**); Table S2. ^1^H and ^13^C NMR data of 9-oxonerolidol (**2**); Table S3. ^1^H and ^13^C NMR data of chiliadenol B (**3**).
