# Supplementary material for: Farnesane-Type Sesquiterpenoids with Antibiotic Activity from Chiliadenus lopadusanus
Source: Antibiotics (Basel). 2021 Feb 2;10(2):148. doi: 10.3390/antibiotics10020148 (PMC7913021; doi:10.3390/antibiotics10020148)
Supplement: Supplementary file 1 [file antibiotics-10-00148-s001.pdf]

SUPPORTING INFORMATION

Farnesane-type sesquiterpenoids with antibiotic activity from *Chiliadenus lopadusanus*

Marco Masi[1,†], Emanuela Roscetto[2,†], Alessio Cimmino[1,*], Maria Rosaria Catania[2], Giuseppe Surico[3] and Antonio Evidente[1]

1 Dipartimento di Scienze Chimiche, Università di Napoli Federico II, Complesso Universitario Monte S. Angelo, Via Cintia 4, 80126 Napoli, Italy; marco.masi@unina.it (M.M.); evidente@unina.it (A.E.)

2 Dipartimento di Medicina Molecolare e Biotecnologie Mediche, Università di Napoli Federico II, Via Pansini 5, 80131 Napoli, Italy; emanuela.roscetto@unina.it (E.R.); mariarosaria.catania@unina.it (M.R.C.)

3 Dipartimento di Scienze e Tecnologie Agrarie, Alimentari, Ambientali e Forestali, Sez. Patologia vegetale ed entomologia, Università di Firenze, Piazzale delle Cascine 28, 50144 Firenze, Italy; giuseppe.surico@unifi.it

† These authors equally contribute to this work and are listed in alphabetical order

*Correspondence: alessio.cimmino@unina.it ; Tel.: (+39 081 2532126)

Received: date; Accepted: date; Published: date

Abstract: *Chiliadenus lopadusanus* Brullo is an Asteraceae plant species endemic to Lampedusa island, the largest island of the Pelage archipelago. The organic extract of leaves, showing an antibiotic activity against Gram-positive and Gram-negative bacteria, was bioguided and purified affording three main farnesane-type sesquiterpenoids. They were identified by spectroscopic methods (essentially 1D and 2D ^{1}H and ^{13}C NMR and ESIMS data) as the (*E*)-3,7,11-trimethyldodeca-1,6,10-triene-3,9-diol, (*E*)-10-hydroxy-2,6,10-trimethyldodeca-2,6,11-trien-4-one, and (*E*)-10-hydroxy-2,6,10-trimethyl-dodeca-6,11-dien-4-one, commonly named 9-hydroxynerolidol, 9-oxonerolidol, and chiliadenol B, respectively. These three sesquiterpenes, isolated for the first time from *C. lopadusanus*, were tested on important nosocomial pathogens showing antibacterial and antibiofilm activities. This plant could be used as a source to isolate secondary metabolites as potential new antibiotics.

Keywords: *chiliadenus lopadusanus*; sesquiterpenes; antibacterial activity, antibiofilm activity

Supporting Information List

Page 3: **Figure S1**. ^{1}H NMR spectrum of 9-hydroxynerolidol, **1** ($CDCl_3$, 400 MHz).

Page 3: **Figure S2**. ^{13}C NMR spectrum of 9-hydroxynerolidol, **1** ($CDCl_3$, 100 MHz).

Page 4: **Figure S3**. COSY spectrum of 9-hydroxynerolidol, **1** ($CDCl_3$, 400 MHz).

Page 4: **Figure S4**. HSQC spectrum of 9-hydroxynerolidol, **1** ($CDCl_3$, 400/100 MHz).

Page 5: **Figure S5**. HMBC spectrum of 9-hydroxynerolidol, **1** ($CDCl_3$, 400/100 MHz).

Page 5: **Figure S6**. NOESY spectrum of 9-hydroxynerolidol, **1** ($CDCl_3$, 400 MHz).

Page 6: **Figure S7**. ESIMS spectrum of 9-hydroxynerolidol, **1,** recorded in positive modality.

Page 6: **Figure S8**. ^{1}H NMR spectrum of 9-oxonerolidol, **2** ($CDCl_3$, 400 MHz).

Page 7: **Figure S9**. ^{13}C NMR spectrum of 9-oxonerolidol, **2** ($CDCl_3$, 100 MHz).

Page 7: **Figure S10**. COSY spectrum of 9-oxonerolidol, **2** ($CDCl_3$, 400 MHz).

Page 8: **Figure S11**. HSQC spectrum of 9-oxonerolidol, **2** ($CDCl_3$, 400/100 MHz).

Page 8: **Figure S12**. HMBC spectrum of 9-oxonerolidol, **2** ($CDCl_3$, 400/100 MHz).

Page 9: **Figure S13.** NOESY spectrum of 9-oxonerolidol, **2** ($CDCl_3$, 400 MHz).

Page 9: **Figure S14**. ESIMS spectrum of 9-oxonerolidol, **2**, recorded in positive modality.

Page 10: **Figure S8**. ^{1}H NMR spectrum of chiliadenol B, **3** ($CDCl_3$, 400 MHz).

Page 10: **Figure S9**. ^{13}C NMR spectrum of chiliadenol B, **3** ($CDCl_3$, 100 MHz).

Page 11: **Figure S10**. COSY spectrum of chiliadenol B, **3** ($CDCl_3$, 400 MHz).

Page 11: **Figure S11**. HSQC spectrum of chiliadenol B, **3** ($CDCl_3$, 400/100 MHz).

Page 12: **Figure S12**. HMBC spectrum of chiliadenol B, **3** ($CDCl_3$, 400/100 MHz).

Page 12: **Figure S13.** NOESY spectrum of chiliadenol B, **3** ($CDCl_3$, 400 MHz).

Page 13: **Figure S14**. ESIMS spectrum of chiliadenol B, **3**, recorded in positive modality.

Page 13: **Table S1.** ^{1}H and ^{13}C NMR data of 9-hydroxynerolidol (**1**).

Page 14: **Table S2.** ^{1}H and ^{13}C NMR data of 9-oxonerolidol (**2**).

Page 14: **Table S3.** ^{1}H and ^{13}C NMR data of chiliadenol B (**3**).

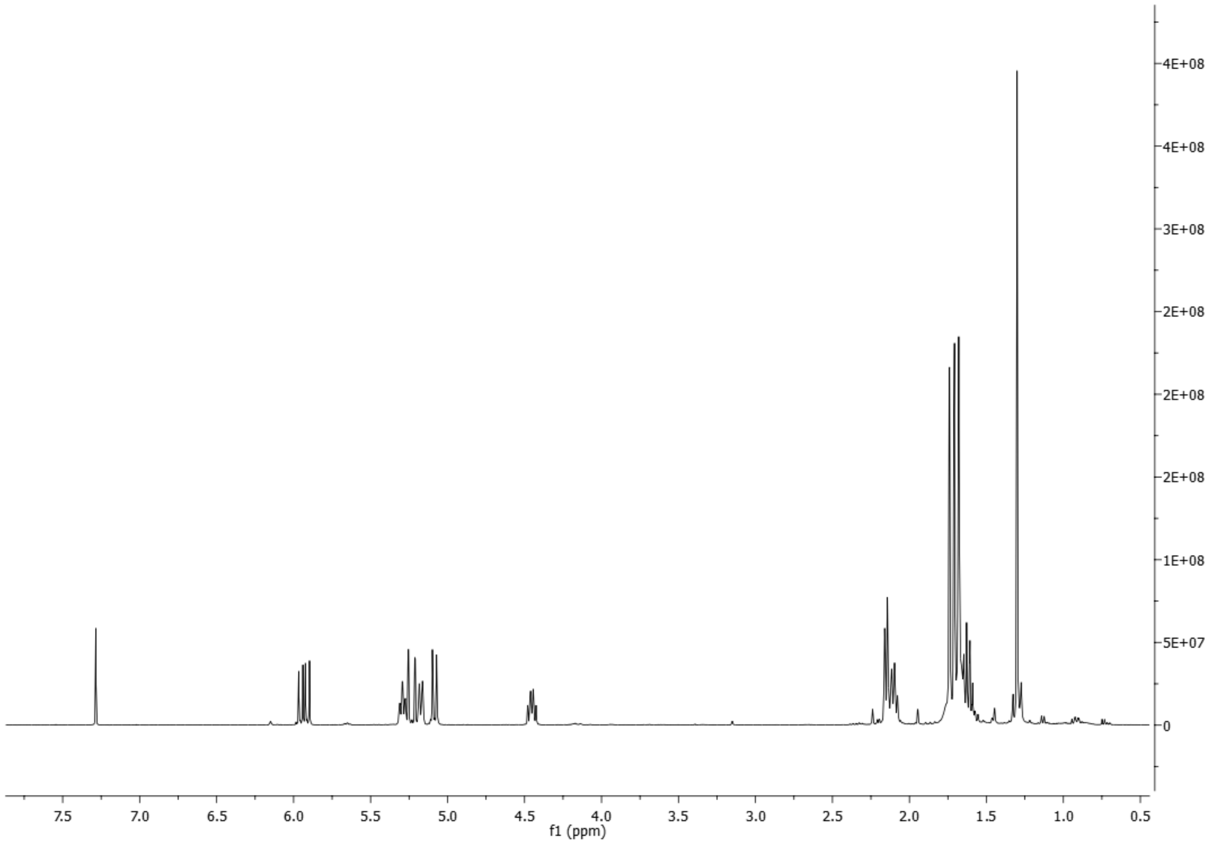

Figure S1. ^{1}H NMR spectrum of 9-hydroxynerolidol, **1** ($CDCl_3$, 400 MHz).

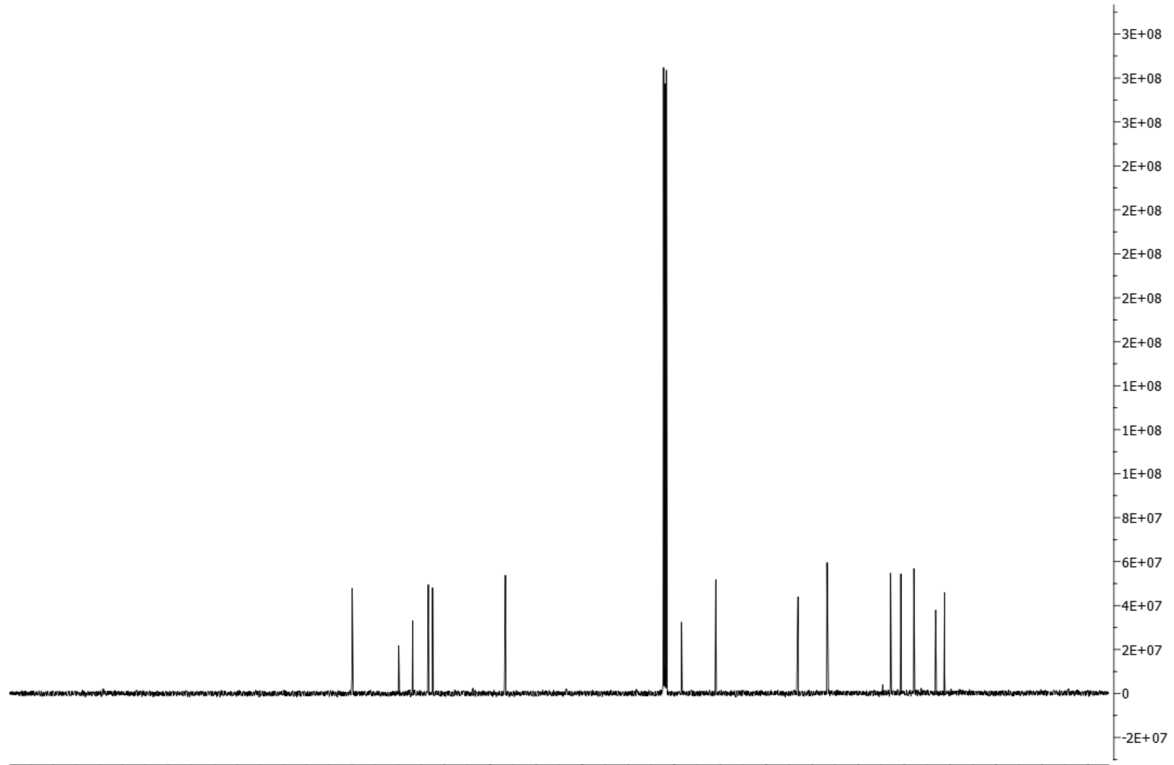

Figure S2. ^{13}C NMR spectrum of 9-hydroxynerolidol, **1** ($CDCl_3$, 100 MHz).

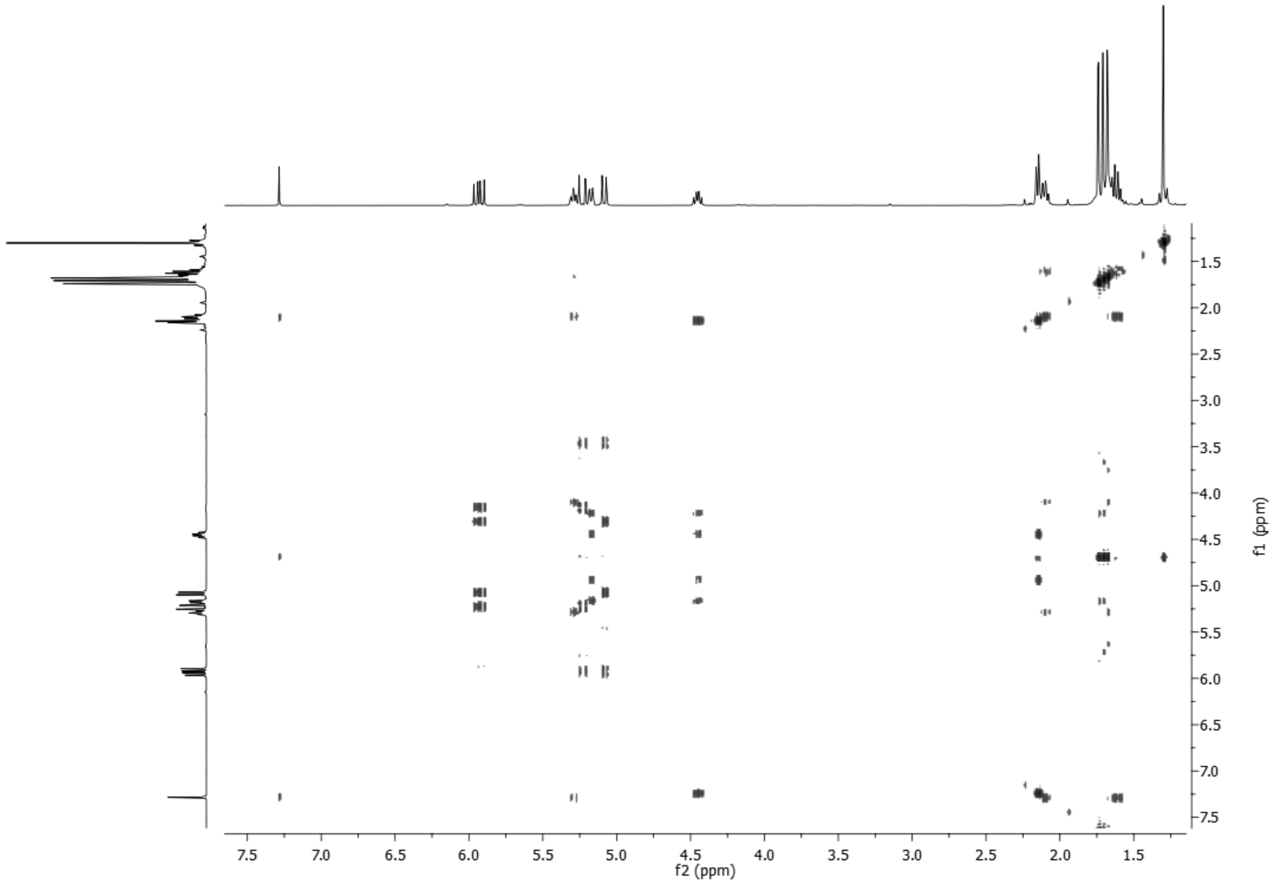

Figure S3. COSY spectrum of 9-hydroxynerolidol, **1** ($CDCl_3$, 400 MHz).

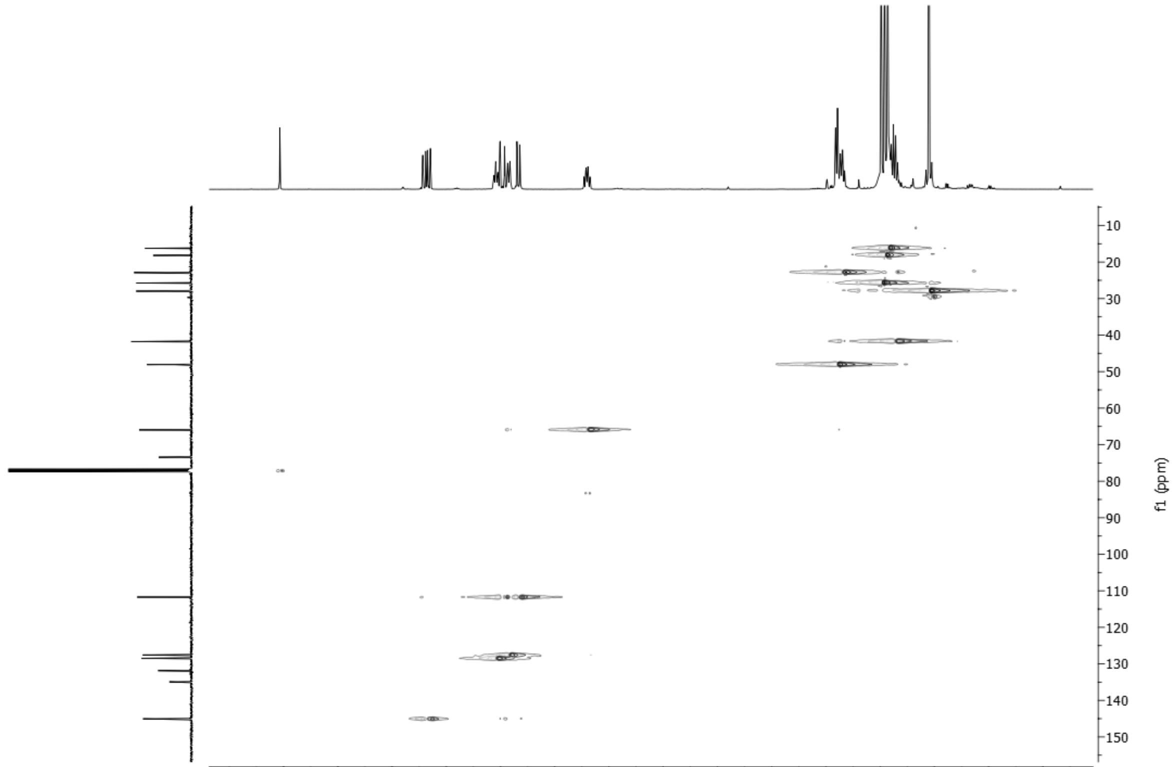

Figure S4. HSQC spectrum of 9-hydroxynerolidol, **1** ($CHCl_3$, 400/100 MHz).

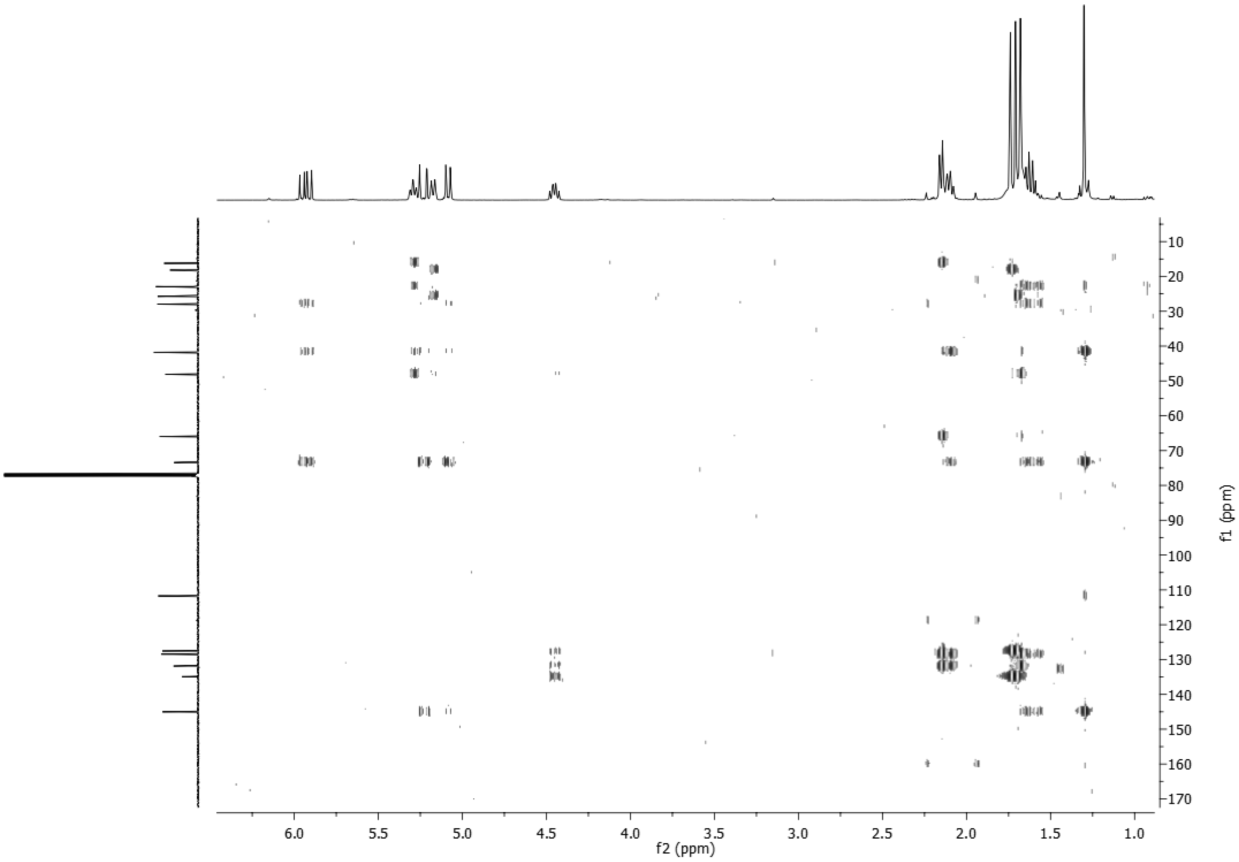

Figure S5. HMBC spectrum of 9-hydroxynerolidol, **1** ($CHCl_3$, 400/100 MHz).

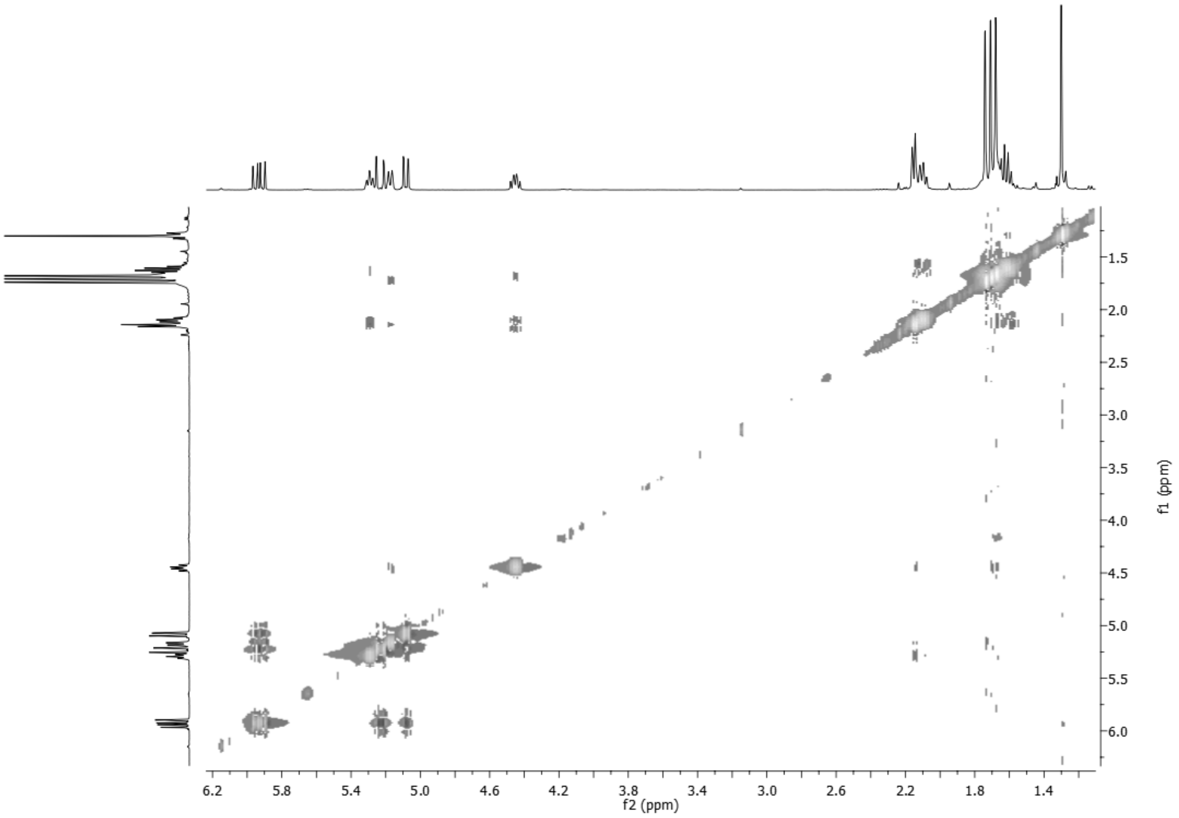

Figure S6. NOESY spectrum of 9-hydroxynerolidol, **1** ($CDCl_3$, 400 MHz).

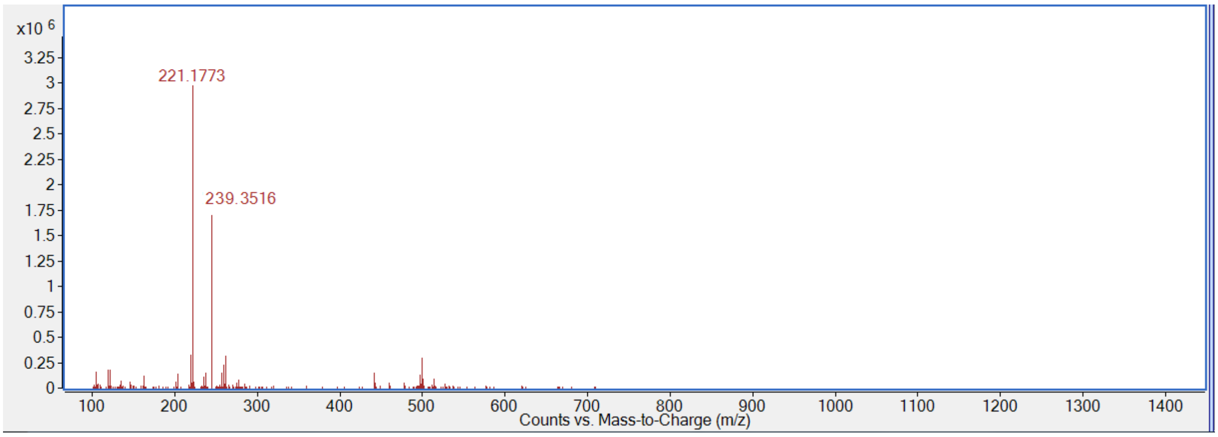

Figure S7. ESIMS spectrum of 9-hydroxynerolidol, **1**, recorded in positive modality.

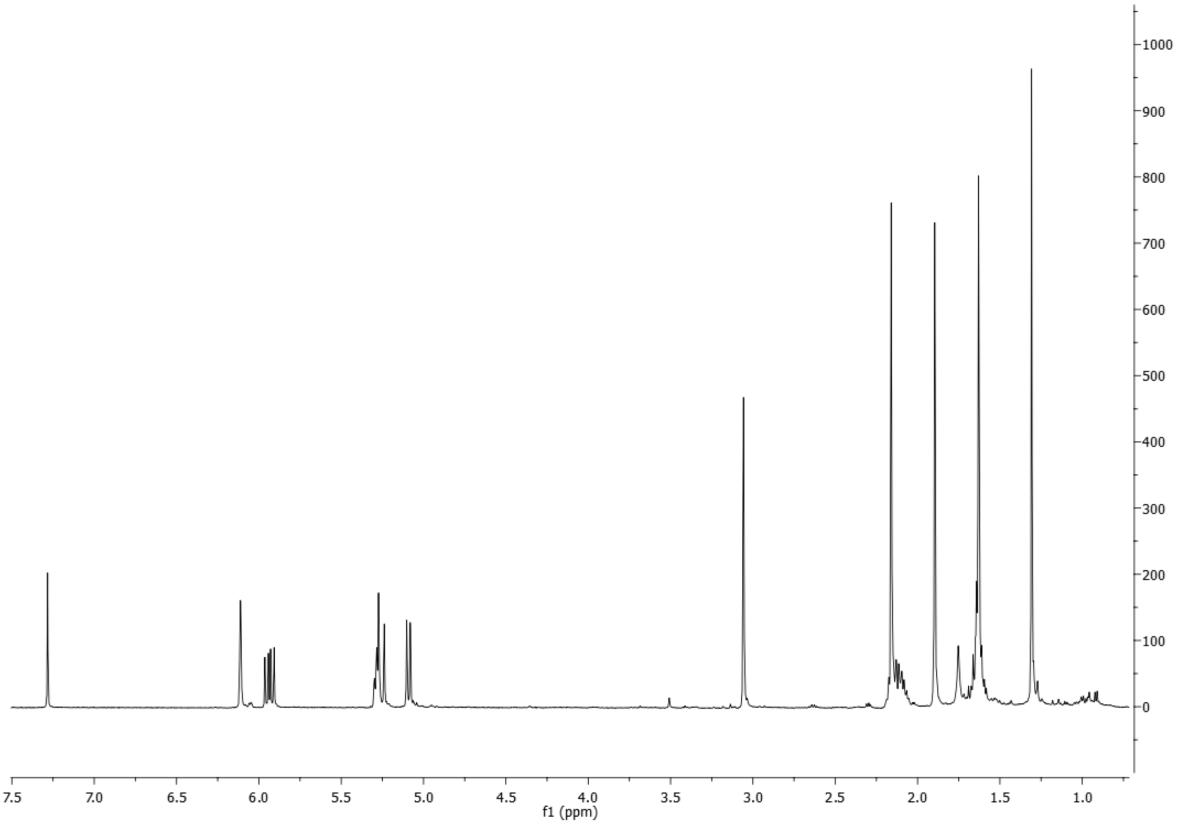

Figure S8. ^{1}H NMR spectrum of 9-oxonerolidol, **2** ($CDCl_3$, 400 MHz).

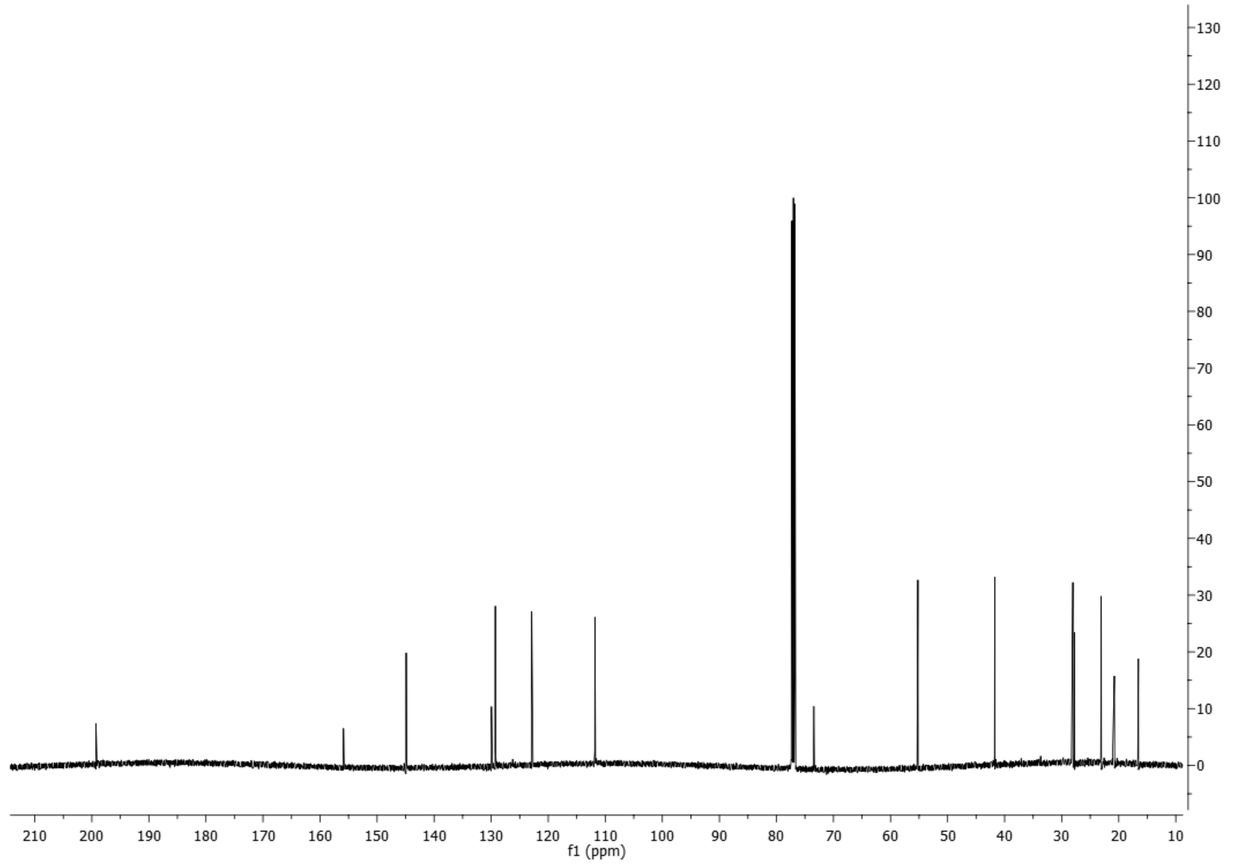

Figure S9. ^{13}C NMR spectrum of 9-oxonerolidol, **2** ($CDCl_3$, 100 MHz).

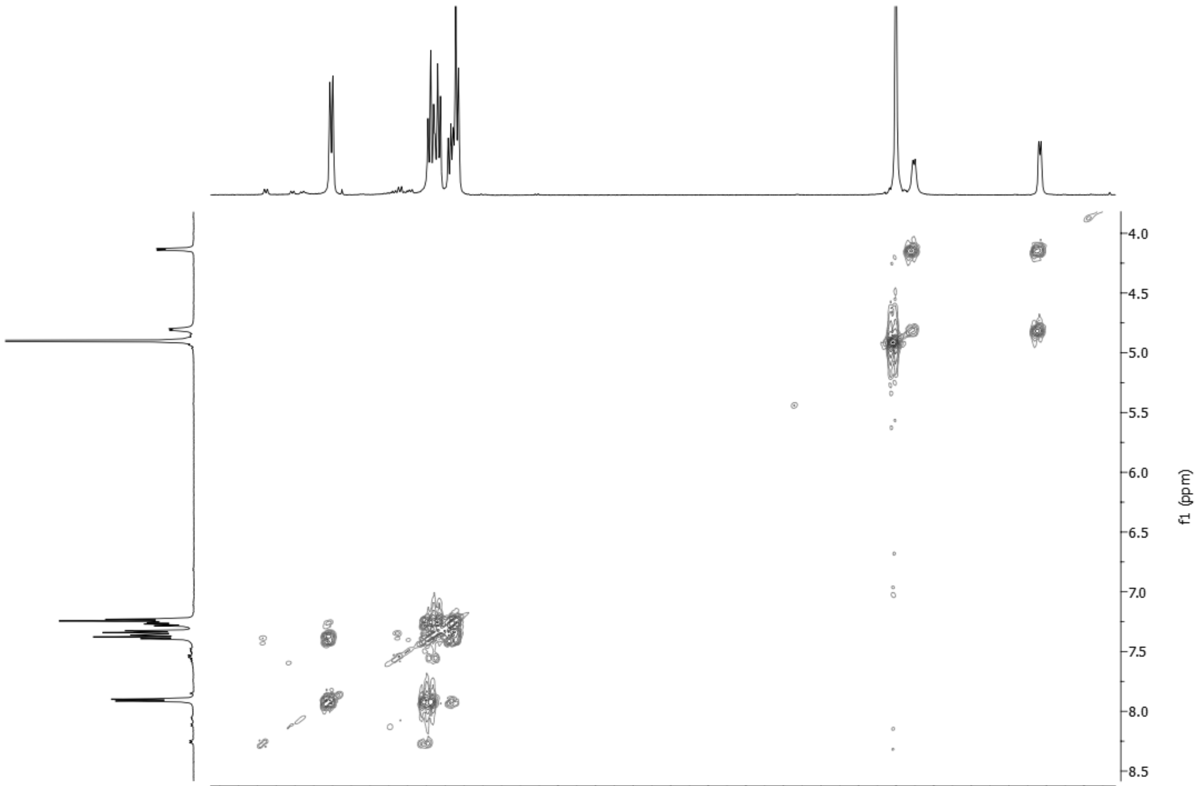

Figure S10. COSY spectrum of 9-oxonerolidol, **2** ($CDCl_3$, 400 MHz).

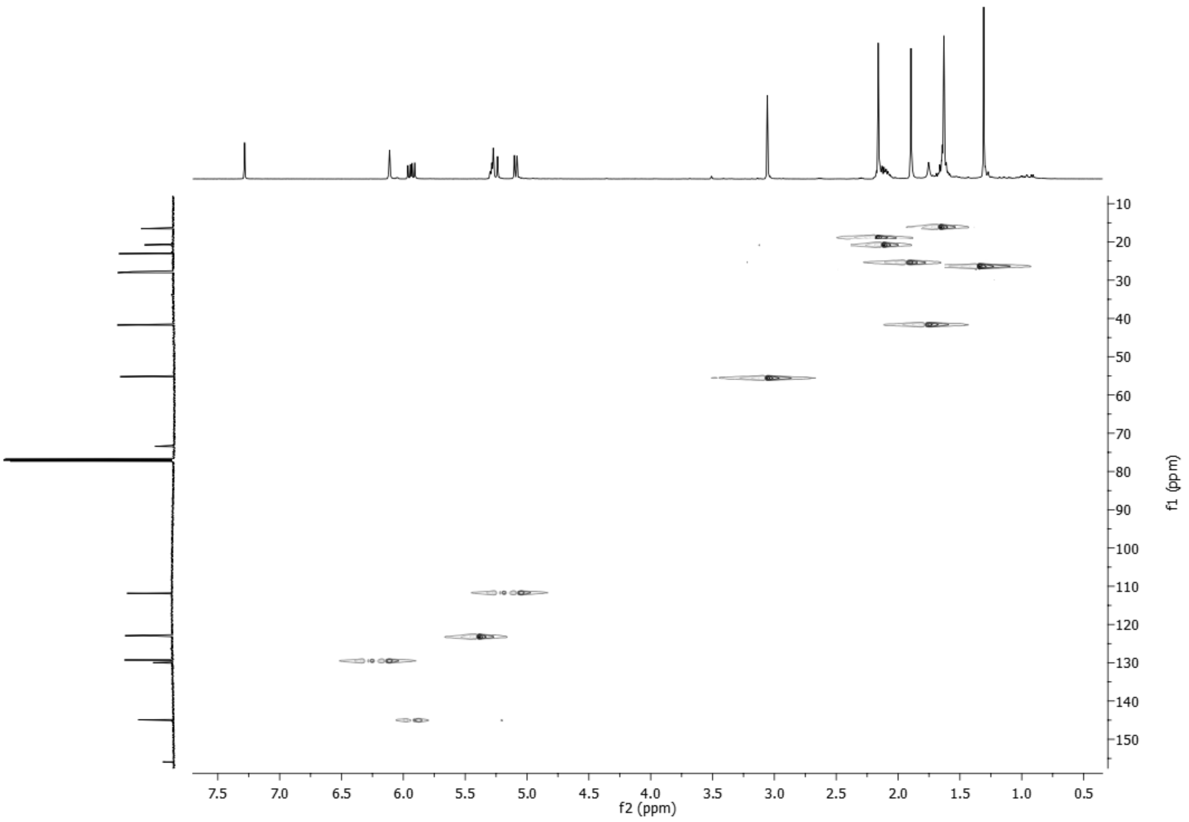

Figure S11. HSQC spectrum of 9-oxonerolidol, **2** ($CDCl_3$, 400/100 MHz).

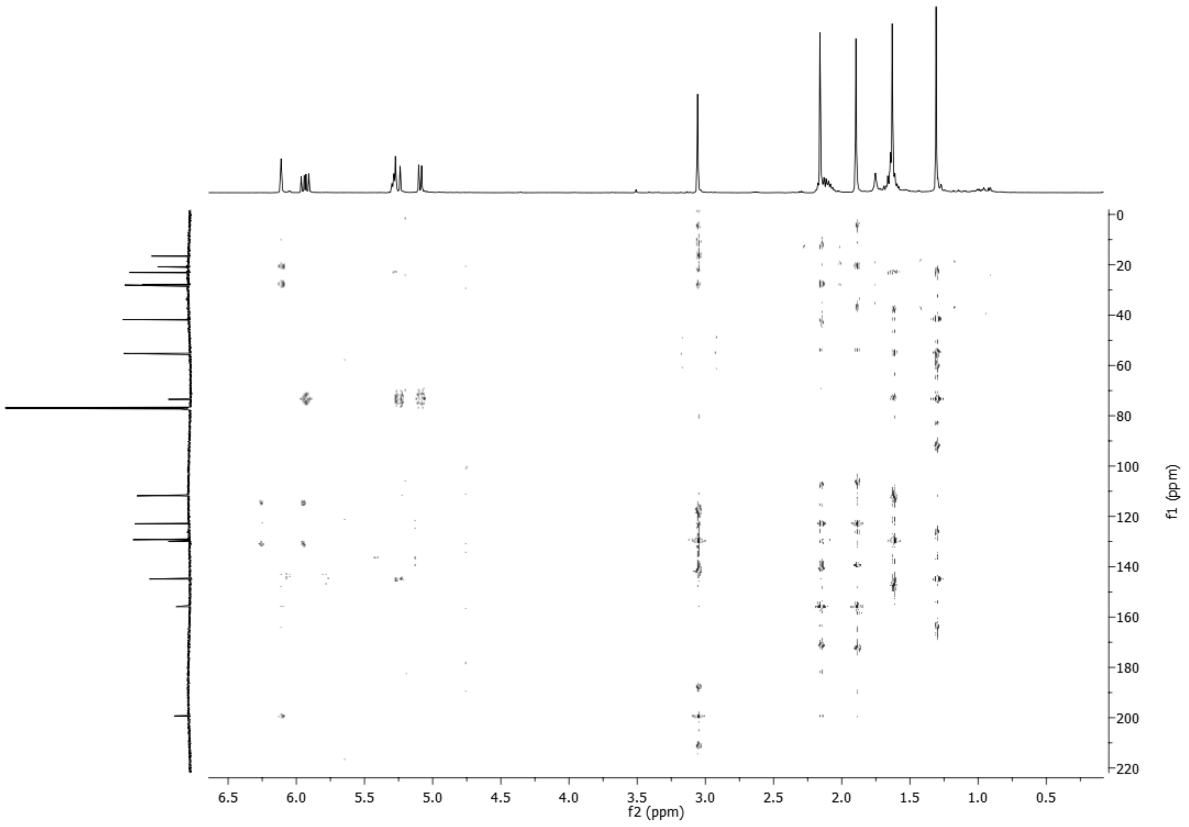

Figure S12. HMBC spectrum of 9-oxonerolidol, **2** ($CDCl_3$, 400/100 MHz).

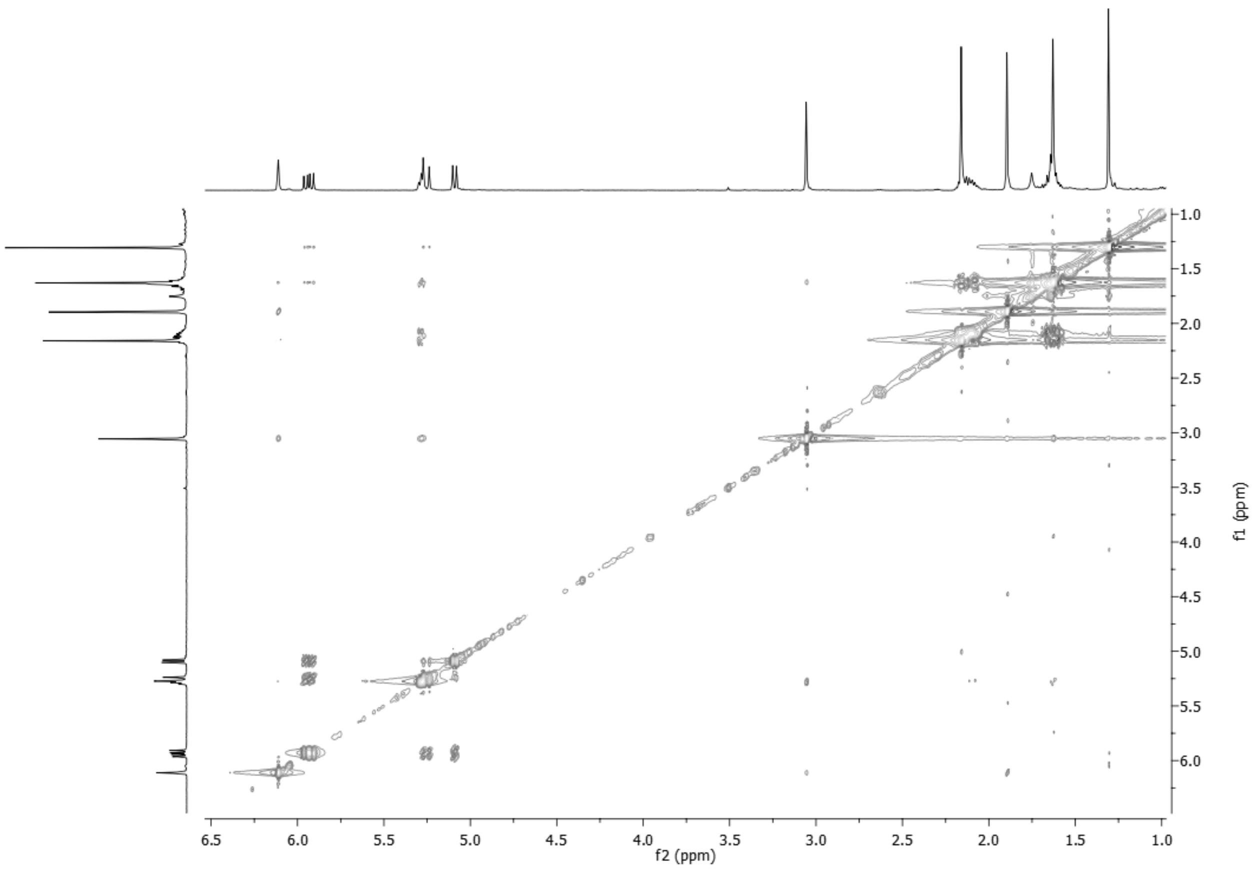

Figure S13. NOESY spectrum of 9-oxonerolidol, **2** ($CDCl_3$, 400 MHz).

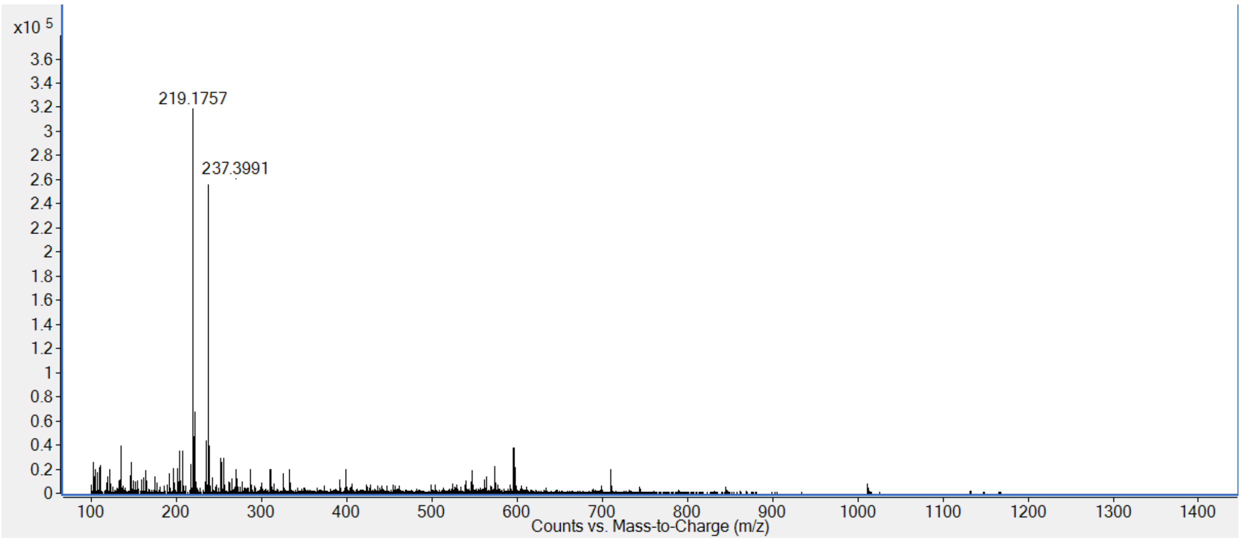

Figure S14. ESIMS spectrum of 9-oxonerolidol, **2**, recorded in positive modality.

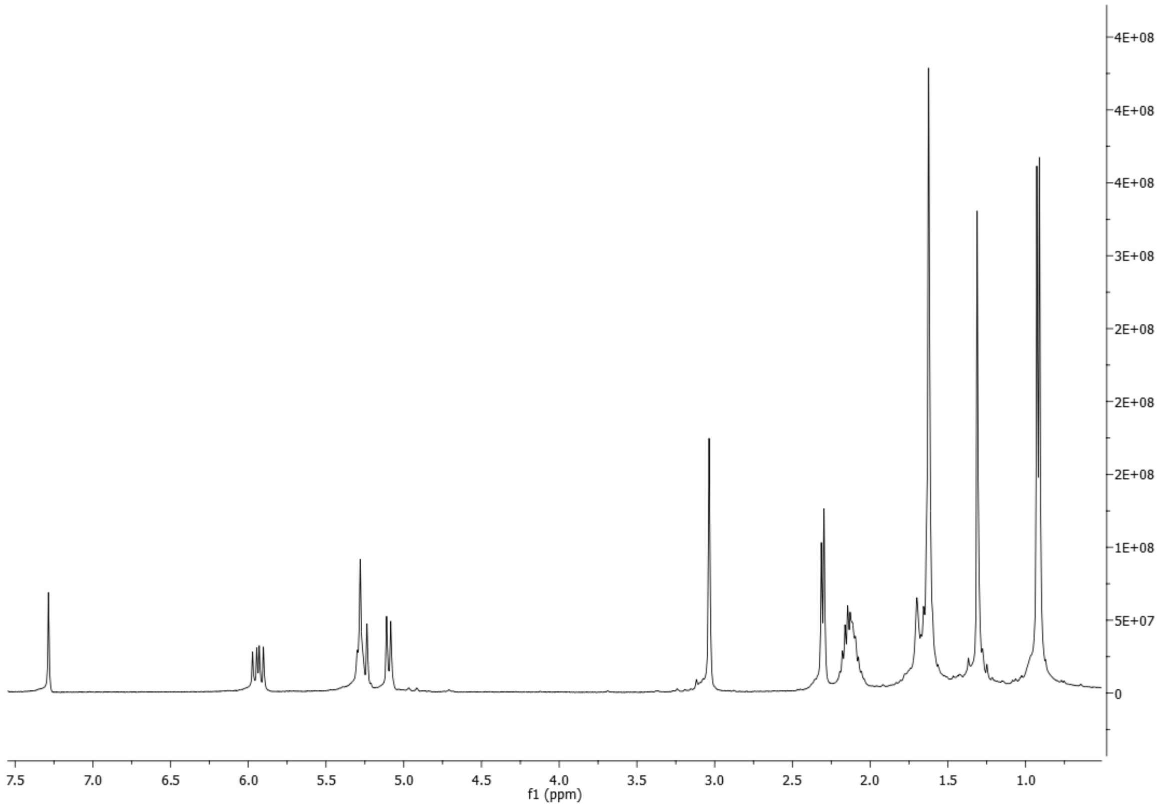

Figure S15. ^{1}H NMR spectrum of chiliadenol B, **3** ($CDCl_3$, 400 MHz).

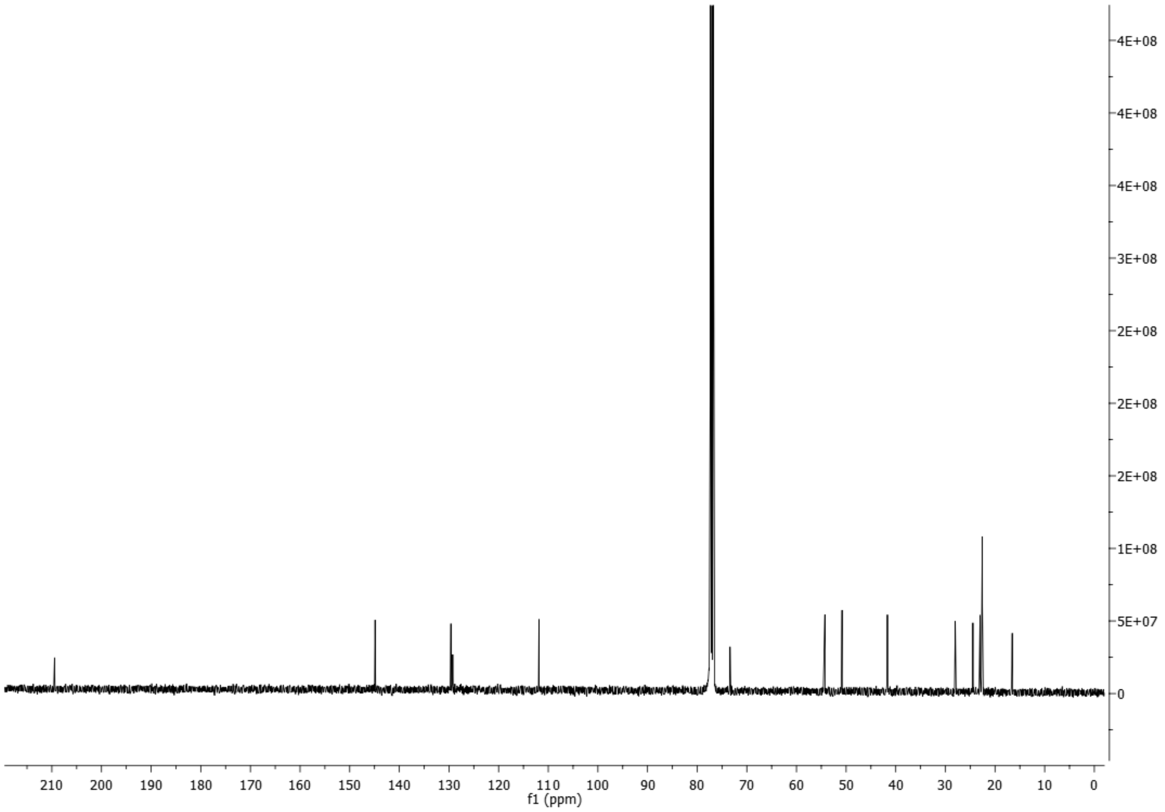

Figure S16. ^{13}C NMR spectrum of chiliadenol B, **3** ($CDCl_3$, 100 MHz).

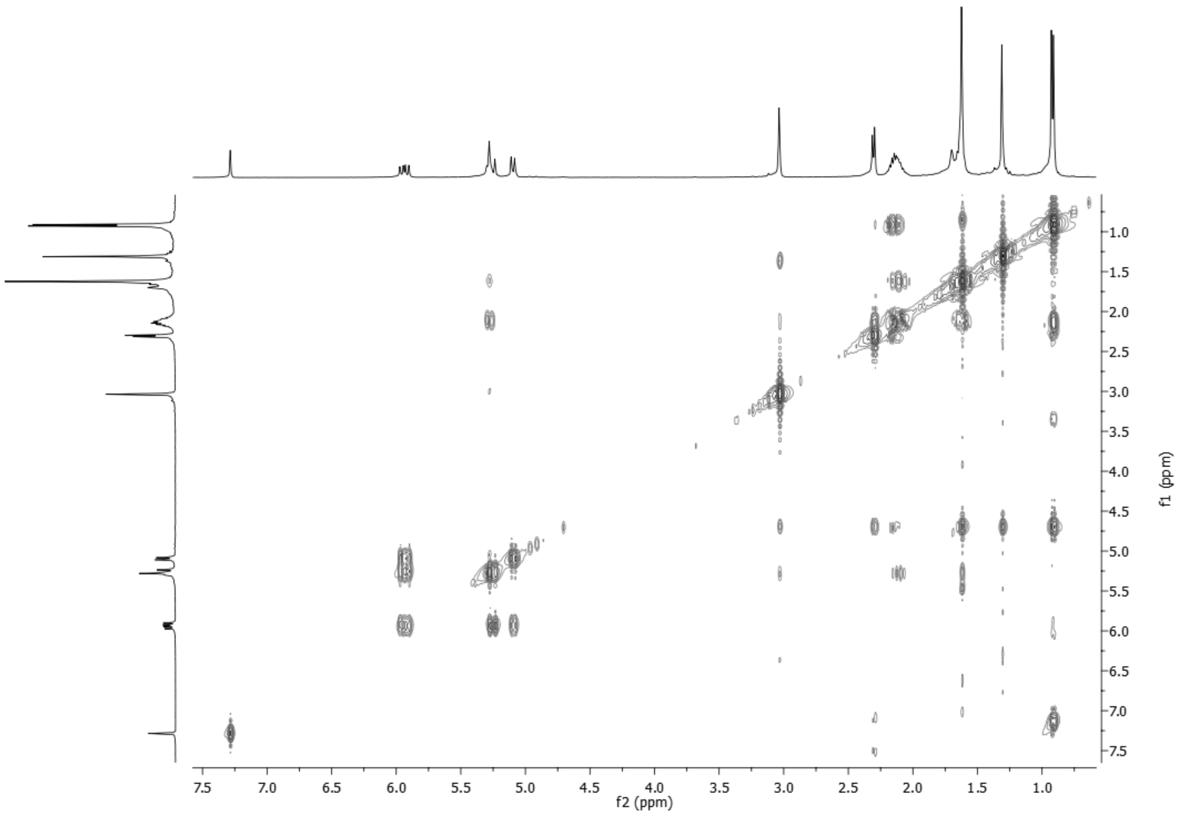

Figure S17. COSY spectrum of chiliadenol B, **3** ($CDCl_3$, 400 MHz).

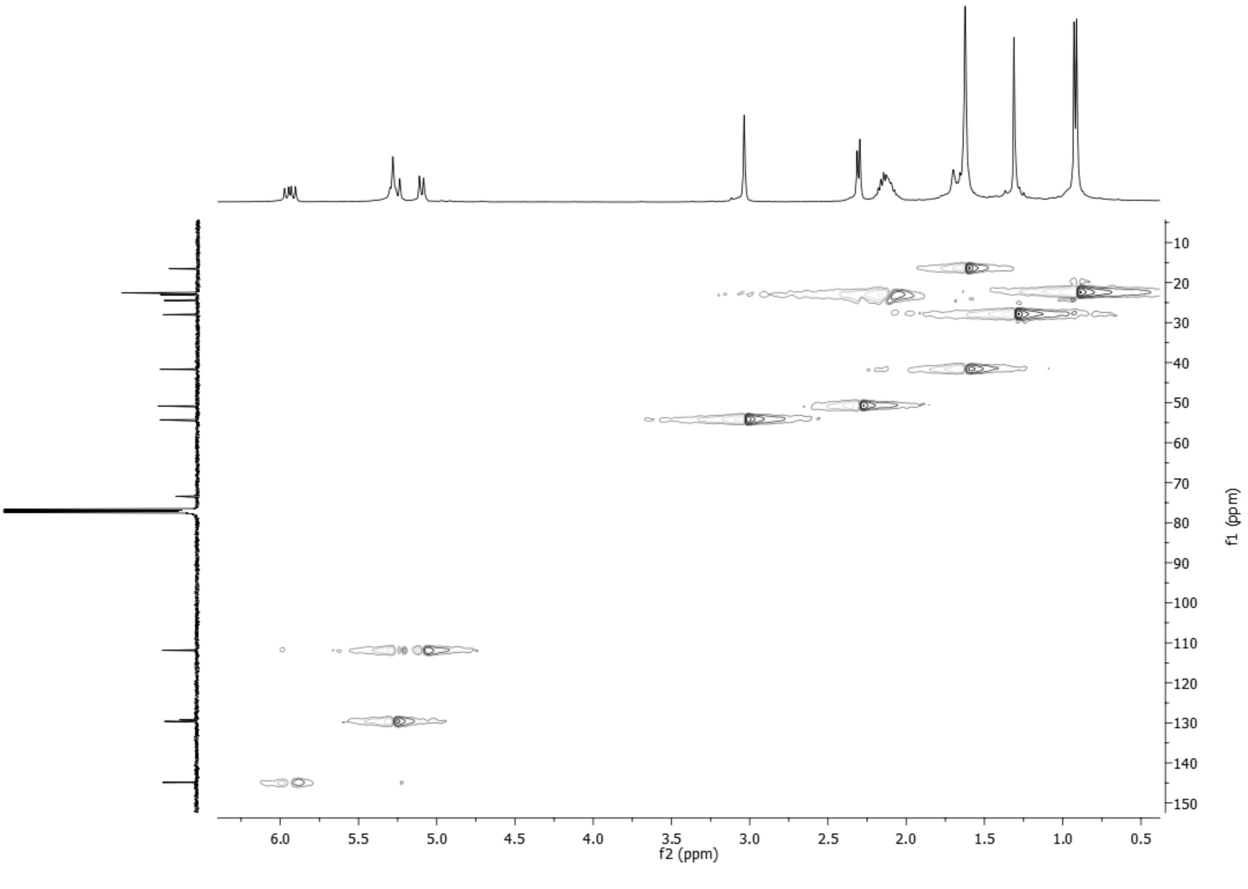

Figure S18. HSQC spectrum of chiliadenol B, **3** ($CDCl_3$, 400/100 MHz).

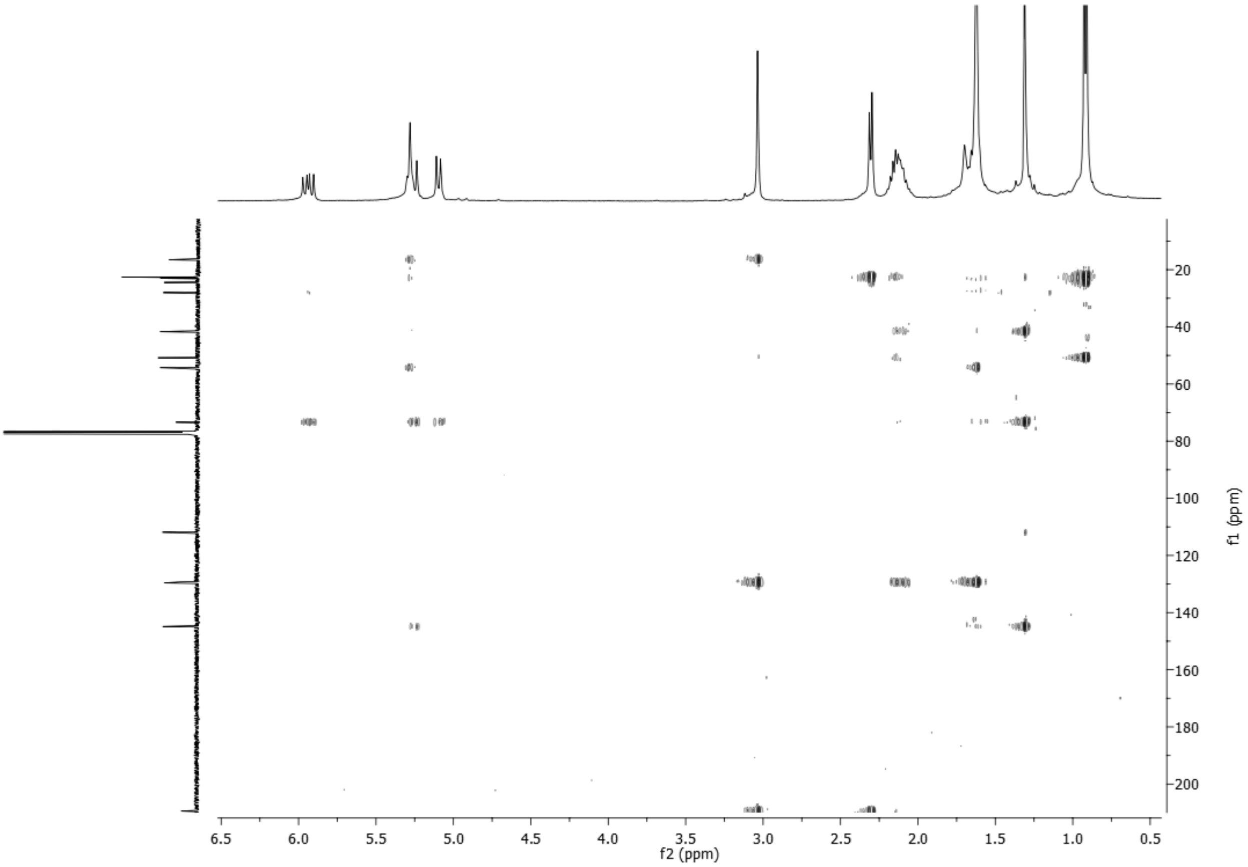

Figure S19. HMBC spectrum of chiliadenol B, **3** ($CDCl_3$, 400/100 MHz).

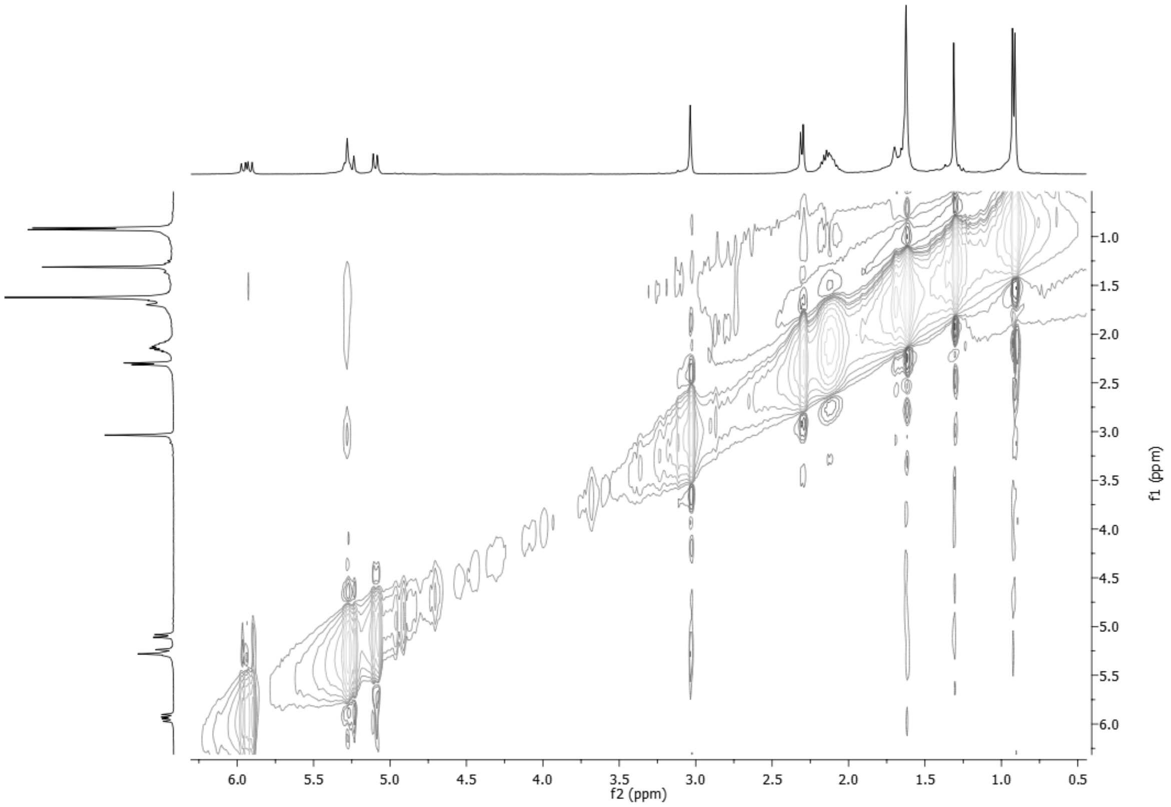

Figure S20. NOESY spectrum of chiliadenol B, **3** ($CDCl_3$, 400 MHz).

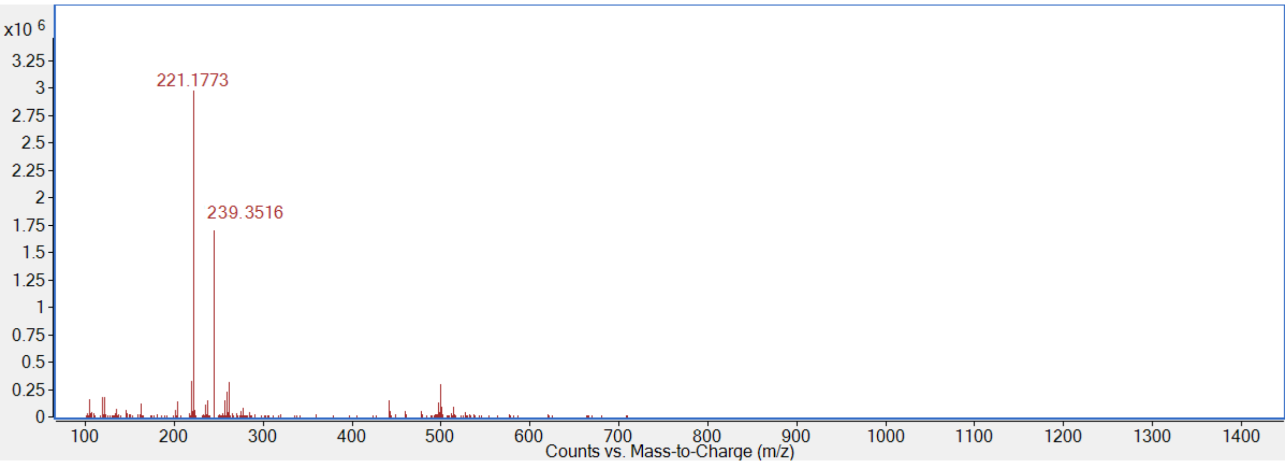

Figure S21. ESIMS spectrum of chiliadenol B, **3** recorded in positive modality.

Table S1. ^{1}H and ^{13}C NMR data of 9-hydroxynerolidol (**1**)[a,b].

Position	δC[c]	δH (J in Hz)	HMBC
1	111.7 t	5.08 dd (10.7, 1.2)	Me-15,
		5.23 dd (17.2, 1.2)	
2	144.9 d	5.93 dd (17.2,10.7)	H-1A, Me-15
3	73.4 s	-	H_2-1
4	41.8 t	2.10 (2H) m	H-2, Me-15
5	22.9 t	1.60 (2H) m	
6	127.5 d	5.29 br t (7.3)	H_2-4, H_2-8, Me-14
7	134.6 s	-	H-9, Me-14
8	48.1 t	2.02 (2H) m	H-6, Me-14
9	66.0 d	4.66 ddd (11.8, 8.5, 2.7)	
10	128.4 d	5.17 br dd (8.5, 1.3)	H_2-8
11	131.8 s	-	H-9
12[d]	25.7 q	1.74 s	
13 [d]	18.1 q	1.71 s	
14	16.2 q	1.68 s	
15	27.9 q	1.30 s	

[a] The chemical shifts are in δ values (ppm) from TMS. [b] 2D ^{1}H, ^{1}H (COSY) ^{13}C, ^{1}H (HSQC) NMR experiments delineated the correlations of all the protons and the corresponding carbons. [c] Multiplicities were assigned by the DEPT spectrum. [d] The signals of the two methyl groups could be exchanged.

Table S2. ^{1}H and ^{13}C NMR data of 9-oxonerolidol (**2**)[a,b].

Position	δCc	δH (J in Hz)	HMBC
1	111.5 t	5.09 br d (10.5)	
		5.26 br d (17.5)	
2	144.5 d	5.94 dd (10.5, 17.5)	H-1A, H-4, H-15
3	73.0 s	-	H_2-1, H-2
4	41.3 t	1.62 (2H) m	Me-15
5	22.9 t	2.10 (2H) m	
6	122.4 d	5.29 br t (7.0)	
7	129.9 s	-	
8	55.3 t	3.06 (2H) s	Me-14,
9	199.2 s	-	H_2-8, H-10
10	129.1 d	6.11 s	H_2-8
11	155.7 s	-	
12[d]	27.5 q	1.90 s	Me-13
13[d]	20.4 q	2.16 s	Me-12
14	16.3 q	1.63 s	H_2-8
15	27.9 q	1.31 s	

[a] The chemical shifts are in δ values (ppm) from TMS. [b] 2D ^{1}H, ^{1}H (COSY) ^{13}C, ^{1}H (HSQC) NMR experiments delineated the correlations of all the protons and the corresponding carbons. [c] Multiplicities were assigned by the DEPT spectrum. [d] The signals of the two methyl groups could be exchanged.

Table S3. ^{1}H and ^{13}C NMR data of chiliadenol B (**3**)[a,b].

Position	δCc	δH (J in Hz)	HMBC
1	111.8 t	5.10 br d (10.7)	Me-15
		5.26 br d (17.3)	
2	144.8 d	5.94 dd (10.7, 17.3)	H-1A, H_2-4, Me-15
3	73.0 s	-	H_2-1, H-2, Me-15
4	41.4 t	1.67 (2H) m	H_2-5, Me-15
5	22.7 t	2.10 (2H) m	H-6, H_2-4
6	129.4 d	5.28 t (7.3)	H_2-8, H_2-5, H_2-4
7	128.9 s	-	H-6, Me-14
8	54.0 t	3.04 (2H) s	H-6, Me-14
9	209.4 s	-	H_2-8, H_2-10, Me-14
10	50.4 t	2.30 d (2H) (6.7)	H-11, Me-12/Me-13
11	24.2 t	2.14 m	H-10, Me-12/Me-13
12[d]	22.6 q	0.93 br s	H-10, H-11, Me-13
13[d]	22.6 q	0.91 br s	H-10, H-11, Me-12
14	16.3 q	1.62 s	H_2-8, H-6
15	27.9 q	1.31 s	H-2

[a] The chemical shifts are in δ values (ppm) from TMS. [b] 2D ^{1}H, ^{1}H (COSY) ^{13}C, 1H (HSQC) NMR experiments delineated the correlations of all the protons and the corresponding carbons. [c] Multiplicities were assigned by the DEPT spectrum. [d] The carbon signals of these two methyl groups are overlapped, while their proton could be exchanged.
